# β-Glucosylceramide From Allergic Mothers Enhances Offspring Responsiveness to Allergen

**DOI:** 10.3389/falgy.2021.647134

**Published:** 2021-02-25

**Authors:** Matthew T. Walker, Ryan P. Ferrie, Aki Hoji, Lindsay M. Schroeder-Carter, Jacob D. Cohen, Ronald L. Schnaar, Joan M. Cook-Mills

**Affiliations:** ^1^Allergy/Immunology Division, Northwestern University School of Medicine, Chicago, IL, United States; ^2^Departments of Pediatrics and Microbiology and Immunology, Herman B Wells Center for Pediatric Research, Indiana University School of Medicine, Indianapolis, IN, United States; ^3^Departments of Pharmacology and Neuroscience, Johns Hopkins University School of Medicine, Baltimore, MD, United States

**Keywords:** allergy, β-glucosylceramide, dendritic cell, eosinophils, fetal liver, lipidomics, maternal, neonate

## Abstract

In animals and humans, offspring of allergic mothers have increased responsiveness to allergen and the allergen-specificity of the offspring can be different than that of the mother. In our preclinical models, the mother's allergic responses influence development of the fetus and offspring by elevating numbers of cells in dendritic cell subsets. A major question is the identity of maternal factors of allergic mothers that alter offspring development of responsiveness to allergen. Lipids are altered during allergic responses and lipids are transported to the fetus for growth and formation of fetal membranes. We hypothesized that pro-inflammatory lipids, that are elevated in allergic mothers, are transported to the fetus and regulate fetal immune development. We demonstrate in this report that there was a significant 2-fold increase in β-glucosylceramides (βGlcCer) in allergic mothers, the fetal liver and her offspring. The βGlcCer were transported from mother's plasma, across the placenta, to the fetus and in breastmilk to the offspring. Administration of βGlcCer to non-allergic mothers was sufficient for offspring responses to allergen. Importantly, maternal administration of a clinically relevant pharmacological inhibitor of βGlcCer synthase returned βGlcCer to normal levels in the allergic mothers and her offspring and blocked the offspring increase in dendritic cell subsets and offspring allergen responsiveness. In summary, allergic mothers had increased βGlcCer that was transported to offspring and mediated increases in offspring DCs and responsiveness to allergen. These data have a significant impact on our understanding of mechanisms for development of allergies in offspring of allergic mothers and have the potential to lead to novel interventions that significantly impact risk for allergic disease early in life.

## Introduction

The prevalence of allergic diseases and asthma have dramatically increased in the last 40 years (1–3). In animals and humans, offspring of allergic mothers have increased responsiveness to allergen (4–11). Most reports for child asthma indicate an association with maternal rather than paternal asthma (4). In humans (4) and mice (5–11), the allergen that the offspring responds to is not necessarily the same allergen that induces allergic responses by the mother. The offspring of allergic mothers, that do not receive an allergen challenge, do not have lung eosinophilia and do not have airway hyperresponsiveness, indicating that pup lung responses are induced by allergen challenge of the offspring of allergic mothers (5). The duration of the maternal effect on induction of offspring allergen hyperresponsiveness for development of lung eosinophilia in mice is sustained up to at least 12 weeks after birth, although the magnitude of response to allergen diminishes after 6 weeks (6). The heightened responses to allergen by offspring of allergic mothers is mediated by alterations in dendritic cells of offspring of allergic mothers (4, 12). Briefly, DCs from pups of allergic mothers have enhanced allergen presentation function (12). Moreover, in mice, transfer of dendritic cells (DCs) from neonates of allergic mothers (but not macrophages) to recipient neonates from non-allergic mothers transfers the enhanced allergic responsiveness to the recipient neonates (4, 12). This indicates that DCs of offspring of allergic mothers are sufficient for the allergen responsiveness. We have shown that the fetal liver and offspring of these allergic mothers also have increased numbers of distinct subsets of CD11b+ CD11c+ DCs without altering CD103+ DCs (13, 14). These lung DCs of OVA-challenged pups of allergic mothers are of a Th2 phenotype as demonstrated by increased numbers of IRF+ DCs (14).

The maternal mediators of allergic mothers that increase DCs and allergen responsiveness in the offspring are not known. Allergen, IgE, IL-4, and IL13 of the mother do not pass to the fetus from the mother (15, 15–17). It has been suggested that cortisol in allergic female mice may contribute to the offspring's responsiveness (18). Interestingly, lipids, which are pro- or anti-inflammatory, are actively transported across the placenta (19). Concentrations of some lipid metabolites are altered during allergic inflammation in humans and mice (20–25). Lipid metabolites regulate DC differentiation/function (26–29) and DCs mediate initiation of allergic disease (4, 12). Furthermore, maternal lipids (30–32) are also present in milk and may regulate development of the immune system in the neonate. In studies of cross-fostered offspring, milk of allergic mothers is sufficient for enhanced allergic responsiveness of offspring (16). Thus, lipids of allergic mothers may regulate inflammation, lipids circulate through the liver, and the fetal liver is the site of hematopoiesis/immune system development.

The lipid metabolites sphingolipids and ceramides have been reported to increase with allergy (20), are transported on HDL/LDL (33, 34), and can regulate DC development and function (20, 35). DC development and function can be regulated by sphingosine 1-phosphates (S1P) (35), S1P regulates allergic inflammation in adults (20) and S1P is transported on HDL (36). Also, dihydro-S1P has been described as inhibitory for TGF-β signaling, suggesting that dihydro-S1P is pro-inflammatory (37).

Besides S1P, β-monohexosylceramides (βHexCer) have pro-inflammatory properties in allergy (20, 21) and in other models of inflammation (20, 21, 37, 38). β-monohexosyl-ceramide (βHexCer) in peripheral tissues is primarily β-monoglucosylceramide (βGlcCer) and β-monogalactosylceramide (βGalCer). βGlcCer is generated by glycosylation of ceramide by the rate limiting enzyme glucosylceramide synthase (39, 40). Administration of βGlcCer enhances IL-4 in graft vs. host disease (38). Studies suggest that immunomodulatory effects of βGlcCer are dependent upon DCs (38) and we reported that DCs are elevated in offspring of allergic mothers (13, 14). Although one report suggests that high doses of βGlcCer, repeatedly administered to the lungs of mice, reduces allergic inflammation (41), it is not known whether endogenous changes in systemic levels of βGlcCer regulate development of allergy in offspring of allergic mothers.

In this report, we demonstrated that βGlcCer was elevated in allergic mothers, was transported to the offspring, and mediated the enhanced offspring responsiveness to allergens. This was specific to βGlcCer because in offspring of allergic mothers, there was no increase in ceramides, sphingomyelins, sphingosines, sphingosine 1-phosphate, dihydrosphingosine and dihydrosphingosine 1-phosphate.

## Methods

### Animals

Adult C57BL/6 female and male mice were from Jackson Laboratory, Bar Harbor, Maine.

#### OVA and Inhibitor Administration and Analysis of Inflammation

C57BL/6 female mice were maintained on chow diet. The mice were sensitized by intraperitoneal injection (200 μl) of OVA grade V (5 μg)/alum (1 mg) or saline/alum (1 mg) on days 0 and 7 (4–6). The mice were exposed to nebulized saline or 3% (w/v) OVA in saline for 15 min on three consecutive days at 8, 12, and 16 weeks of age and then mated. The nebulization was performed using a DeVilBiss Pulmo-Aide Compressor with Disposable Nebulizer attached with tygon tubing to a polypropylene chamber with tubing adaptor inserted into the side of a chamber. The chamber was a square clear 19 cup Rubbermaid Servin' Saver container with lid in which we inserted an inlet and an outlet 3/8 inch hole on opposite on sides of chamber to maintain normal ambient pressure in the chamber during nebulization. A piece of lab tape was loosely placed over the outlet port such that it still allowed airflow out of the chamber. Mice were placed in the chamber for nebulization. Pups were maintained in a single layer during nebulization by an occasional light jiggle of the chamber. In some experiments, βGlcCer was administered to pregnant and nursing dams as indicated in the figures. Tissues were collected at gestational day 18 or in postnatal day 13 pups. At gestational day 18, the mice were euthanized and the plasma, lung, liver, placenta and fetal livers were collected. Alternatively, 3 days old pups were *sub-optimally* sensitized by treating with only one 50 μl i.p. injection (rather than two injections) of 5 μg OVA/1 mg alum (5–7). At 10, 11, and 12 days old, the pups were challenged for 15 min with 3% OVA. To inhibit β-glucosylceramide synthase in the groups indicated in the figures, the mothers received daily subcutaneous injections starting on gestational day 5 (GD5) through postnatal day 13 (PND13) of 10 mg (1R,2R)-1-phenyl-2-hexadecanoylamino-3-pyrrolidino-1-propanol (P4rr) or 1S,2S inactive stereoisomer (P4ss) per kg mouse per day. The P4rr and Prss were in an emulsion [ethoxylated castor oil which is PEG35, 20% ethanol, 1% benzyl alcohol (42, 43)]. Injections were rotated at three subcutaneous injection sites on the back. P4rr and P4ss were synthesized by Dr. David Meyers, Synthetic Core Facility, Johns Hopkins University School of Medicine.

At 13 days old, the pups were weighed, euthanized and tissues collected. Pup bronchoalveolar lavage (BAL) cells were stained and counted as previously described (44). OVA-specific IgE was determined by ELISA as previously described (45). IL-5, CCL11, and CCL24 in BAL were measured by ELISA (Raybiotech). For blood eosinophils, peripheral blood was collected in heparin tubes and blood eosinophils were counted in a 1:10 dilution in Discomb stain (one part acetone, one part 2% aqueous eosin, and eight parts distilled water) (46, 47). The eosin positive eosinophils were counted using a hemacytometer (catalog #0267110, Fisher Scientific) (48).

#### Lipid Measurement by Mass Spectrometry

Lipidomics were performed at the Lipidomics Core at Virginia Commonwealth University. Internal lipid standards were purchased from Avanti Polar Lipids (Alabaster, AL). Internal standards were added to samples in 10 μL ethanol:methanol:water (7:2:1) as a cocktail of 250 pmol each. Standards for sphingoid bases and sphingoid base 1-phosphates were 17-carbon chain length analogs: C17-sphingosine, (2S,3R,4E)-2-aminoheptadec-4-ene-1,3-diol (d17:1-So); C17-sphinganine, (2S,3R)-2-aminoheptadecane-1,3-diol (d17:0-Sa); C17-sphingosine 1-phosphate, heptadecasphing-4-enine-1-phosphate (d17:1-So1P); and C17-sphinganine 1-phosphate, heptadecasphinganine-1-phosphate (d17:0-Sa1P). Standards for N-acyl sphingolipids were C12-fatty acid analogs: C12-Cer, N-(dodecanoyl)-sphing-4-enine (d18:1/C12:0); C12-Cer 1-phosphate, N-(dodecanoyl)-sphing-4-enine-1-phosphate (d18:1/C12:0-Cer1P); C12-sphingomyelin, N-(dodecanoyl)-sphing-4-enine-1-phosphocholine (d18:1/C12:0-SM); and C12-glucosylceramide, N-(dodecanoyl)-1-β-glucosyl-sphing-4-eine.

For lipid extractions, samples were collected into 13 x 100 mm borosilicate tubes with a Teflon-lined cap (catalog #60827-453, VWR, West Chester, PA). Then 2 mL of CH_3_OH and 1 mL of CHCl_3_ were added along with the internal standard cocktail (250 pmol of each species dissolved in a final total volume of 10 μl of ethanol:methanol:water 7:2:1). The contents were dispersed using an ultra sonicator at room temperature for 30 s. This single phase mixture was incubated at 48°C overnight. After cooling, 150 μl of 1 M KOH in CH_3_OH was added and, after brief sonication, incubated in a shaking water bath for 2 h at 37°C to cleave potentially interfering glycerolipids. The extract was brought to neutral pH with 12 μl of glacial acetic acid, then the extract was centrifuged using a table-top centrifuge, and the supernatant was removed by a Pasteur pipette and transferred to a new tube. The extract was reduced to dryness using a Speed Vac. The dried residue was reconstituted in 0.5 ml of the starting mobile phase solvent for LC-MS/MS analysis, sonicated for ca 15 s, then centrifuged for 5 min in a tabletop centrifuge before transfer of the clear supernatant to the autoinjector vial for analysis.

For LC-MS/MS analyses of sphingoid bases, sphingoid base 1-phosphates, and complex sphingolipids, the compounds were separated by reverse phase LC using a Supelco 2.1 (i.d.) × 50 mm Ascentis Express C18 column (Sigma, St. Louis, MO) and a binary solvent system at a flow rate of 0.5 mL/min with a column oven set to 35°C. The mass spectrometer was a Shimadzu Nexera LC-30 AD binary pump system coupled to a SIL-30AC autoinjector and DGU20A_5R_ degasser coupled to an AB SCIEX 5500 quadrupole/linear ion trap (QTRAP) (SCIEX Framingham, MA) operating in a triple quadrupole mode. Q1 and Q3 was set to pass molecularly distinctive precursor and product ions (or a scan across multiple *m/z* in Q1 or Q3), using N_2_ to collisionally induce dissociations in Q2 (which was offset from Q1 by 30–120 eV); the ion source temperature set to 500°C. Prior to injection of the sample, the column was equilibrated for 0.5 min with a solvent mixture of 95% Mobile phase A1 (CH_3_OH/H_2_O/HCOOH, 58/41/1, v/v/v, with 5 mM ammonium formate) and 5% Mobile phase B1 (CH_3_OH/HCOOH, 99/1, v/v, with 5 mM ammonium formate), and after sample injection (typically 40 μL), the A1/B1 ratio was maintained at 95/5 for 2.25 min, followed by a linear gradient to 100% B1 over 1.5 min, which was held at 100% B1 for 5.5 min, followed by a 0.5 min gradient return to 95/5 A1/B1. The column was re-equilibrated with 95:5 A1/B1 for 0.5 min before the next run.

#### β-Glucosylceramide and β-Galactosylceramide Analysis by LC-MS/MS

Because glucosylceramide and galactosylceramide co-elute by the above LC-MS/MS method, biological samples that contain both can be analyzed by a separate method. Dried samples are dissolved in CH_3_CN/CH_3_OH/H_3_CCOOH (97:2:1) (v,v,v) with 5 mM ammonium acetate. The LC-Si column (Supelco 2.1 × 250 mm LC-Si) is pre-equilibrated with CH_3_CN/CH_3_OH/H_3_CCOOH (97:2:1) (v,v,v) with 5 mM ammonium acetate for 1.0 min at 1.5 mL per min, sample is injected, and the column is isocratically eluted for 8 min. βGlcCer elutes at 2.56 min and βGalCer at 3.12 min using this isocratic normal phase system; however, column age and previous sample load can influence the retention times of HexCer's by this method. Periodic retention time confirmation with internal standards allows the monitoring of column stability and subsequent effectiveness.

#### qPCR

Total RNA was isolated from 50 to 100 mg lung tissue using the QIAGEN RNeasy Fibrous Tissue Mini Kit (catalog #74704). cDNA was prepared using a SuperScript II RNase H-Reverse Transcriptase kit (Invitrogen Corp., catalog #18064014) and analyzed by PCR on an ABI 7300 Thermal Cycler (Applied Biosystems). Taqman probes and Taqman Universal Master Mix were used as directed (Applied Biosystems, catalog #4304437).

#### Flow Cytometry Analysis of Fetal Liver and Pup Lung Dendritic Cells

Following bronchoalveolar lavage of pups, lungs were removed for analysis of dendritic cell types by flow cytometry. In the fetus, hematopoiesis occurs in the fetal livers. Gestational day 18 fetal livers were collected for analysis of fetal liver dendritic cells. Briefly, tissues were minced and suspended in 5 ml of RPMI solution containing 1 mg/ml collagenase D (Roche, catalog #1108885801) and 0.2 mg/ml DNAse I (Roche, catalog # 04716728001) 37°C with agitation. After 1 h, samples were filtered through sterile 70 μm mesh tissue, centrifuged and resuspended in 5% FBS-RPMI solution. Red blood cells (RBCs) were lysed in 1× BD PharmLyse Lysing Buffer (BD Biosciences, catalog #555899) and the cells washed twice in PBS. All centrifugation steps were carried out at 300× g for 5 min. Three million cells were used per sample for immuno-labeling.

The cells were stained for live/dead exclusion in 500 μl PBS containing 0.25 μl of Aqua LIVE/DEAD fixable (Molecular Probes, Invitrogen) for 20 min at room temperature in the dark. Then, Fc receptors were blocked by incubating the cells in 50 μl flow cytometry staining buffer (Biolegend, catalog #420201) with 0.75 μl purified rat anti-mouse CD16/CD32 Mouse BD Fc Block (BD Pharmingen #553142) for 10 min at 4°C.

To prepare the antibodies for immunolabeling, for each sample equivalent, an antibody stock was prepared by adding the following antibodies to 50 μl of flow cytometry buffer: (1) Live/Dead Aqua fluorescent dye (Molecular Probes, Invitrogen #L34957), 0.25 μl per sample; (2) CD45, rat anti-mouse FITC-conjugated, 0.5 mg/ml, clone 30-F11 (Biolegend #103107), 0.31 μl per sample; (3) CD11b, rat anti-mouse PE-CF594-conjugated, 0.2 mg/ml, clone M1/70 (BD Biosciences #562317), 0.05 μl per sample; (4) Ly-6C, rat anti-mouse APC/Cy7-conjugated, 0.2 mg/ml, clone HK1.4 (Biolegend #128025), 0.4 μl per sample; (5) CD11c, Armenian hamster anti-mouse PE/Cy7-conjugated, 0.2 mg/ml, clone N418 (Biolegend #117317), 0.2 μl per sample; (6) I-A/I-E (MHCII), rat anti-mouse PerCP/Cy5.5-conjugated, 0.2 mg/ml, clone M5/114.15.2 (Biolegend #107625), 0.1 μl per sample; (7) CD103, Armenian hamster anti-mouse Brilliant Violet 421-conjugated, clone 2E7 (Biolegend #121421), 0.2 μl per sample; (8) CD317 (PDCA-1), rat anti-mouse APC-conjugated, 0.2 mg/ml, clone 927 (Biolegend #127015), 0.5 μl per sample; (9) CD80, Armenian hamster anti-mouse PE-conjugated, 0.2 mg/ml, clone 16-10A1 (Biolegend #104707), 0.5 μl per sample; (10) CD86, rat anti-mouse Alexa Fluor 700-conjugated, 0.5 mg/ml, clone GL-1 (Biolegend #105023), 0.5 μl per sample. Then 50 μl of the antibody stock was added to the samples that were treated with Fc block. The samples were incubated for 30 min at 4°C in dark. Then the cells were washed twice in 1× PBS.

The cells were examined with a BD LSRII flow cytometer (BD Biosciences). Analysis was performed using FlowJo VX software (TreeStar, Inc.). Compensation was done using FlowJo compensation wizard based on single color control staining of compensation beads (Invitrogen, catalog #01-1111-41). Non-stained controls were used to assess boundaries of live and dead populations. Only live, singlet (based on FSC-H vs. FSC-A gating), hematopoietic (CD45^+^) cells were used for subsequent gating of all populations. Fluorescence Minus One (FMO) staining controls were used as negative controls to identify gates for populations of interest. The following sub-populations of dendritic cells were analyzed: (1) Resident cDCs: CD11b^+^Ly6C^−^CD11c^high^MHCII^high^; (2) CD11b^+^ Alveolar DCs: CD11b^+^Ly6C^−^CD11c^+^MHCII^−^; (3) Inflammatory DCs: CD11b^+^Ly6C^+^CD11c^+^MHCII^+^; (4) Plasmacytoid DCs: CD11b^−^Ly6C^−^CD11c^low^PDCA-1^+^MHCII^−^; (5) Resident CD103^+^ DCs: CD11b^−^Ly6C^−^CD11c^+^CD103^+^MHCII^−^; (6) CD11b^−^ Alveolar DCs: CD11b^−^Ly6C^−^CD11c^+^MHCII^+/−^. The flow cytometry gating strategy was as previously reported (13).

#### Statistics

Data in the figures were analyzed by a one-way ANOVA followed by Tukey's or Dunnett's multiple comparisons test (SigmaStat, Jandel Scientific, San Ramon, CA). For data with two groups, a *T*-test with two sample unequal variance was used. Data in figures are presented as the means ± the standard errors and each group had 8–10 mice. The lipidomics bubble plot was created by R 4.03, and R packages, rstatix (ver. 0.60) for estimation of fold changes and *p*-values (a pairwise Wilcoxson) and ggplot2 (ver. 3.3.2) for visualization of statistical output. Fold changes of lipids are represented by sizes of filled circles and significant (*p* < 0.05) differential increased lipid levels are displayed as red circles. Data include both genders because there were no differences in outcomes by gender (data not shown).

#### Study Approval

The studies were approved by the Indiana University Institutional Review Committee for animals.

## Results

### In Allergic Mothers, β-Monohexosyl Ceramides (βHexCer) Are Increased in Maternal Plasma, Maternal Liver, Placenta, and Fetal Liver

**Figure 1:**
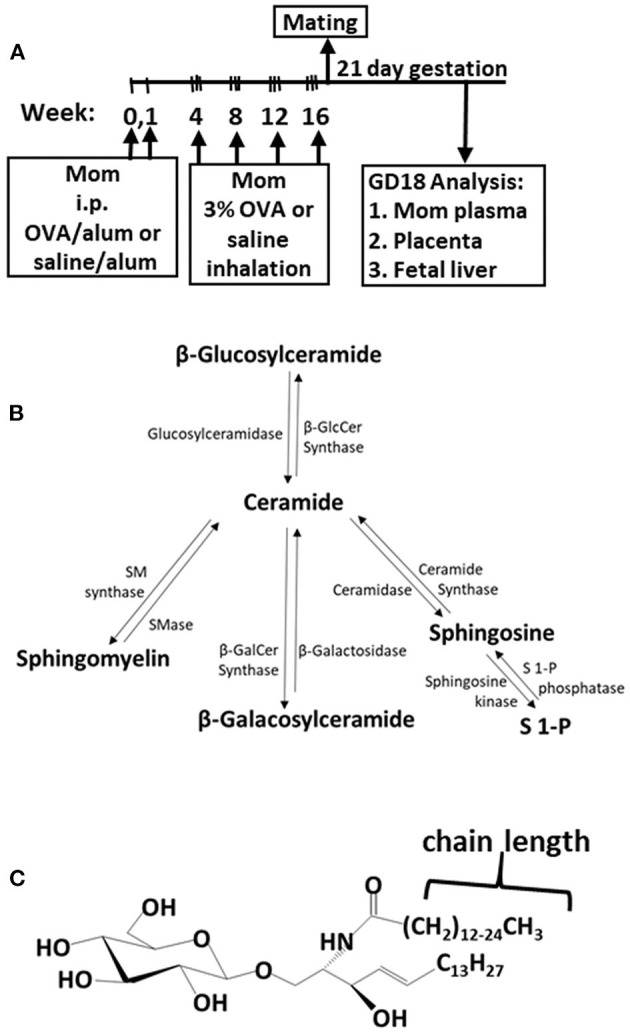
Ceramide pathway and timeline for development of maternal allergy. **(A)** Mothers were sensitized and challenged with OVA or saline and then mated. Tissues were collected on gestational day 18 (GD18) for analysis of ceramides. **(B)** Ceramide metabolite pathway. **(C)** β-monohexosyl ceramide chain length is 14–26 carbons (C14:0 to C26:0) and may contain one double bond, e.g., C18:1. GD18, gestational day 18; i.p., intraperitoneal.

The adult females were treated and mated as in Figure 1A. These mothers develop allergy with allergen-specific IgE as demonstrated in our previous manuscript [Supplementary Figure E6 in reference (49)]. Maternal plasma and placentas on GD18 (Figure 1A) were extracted and examined for ceramides and the ceramide metabolites β-monohexosyl ceramides (βHexCer) which is comprised of βGlcCer and βGalCer (Figure 1B). Tissues were also examined for sphingomyelins and sphingosines. The isoform chain lengths of the ceramide lipids (Figure 1C) were identified by LC-MS/MS.

**Figure 2:**
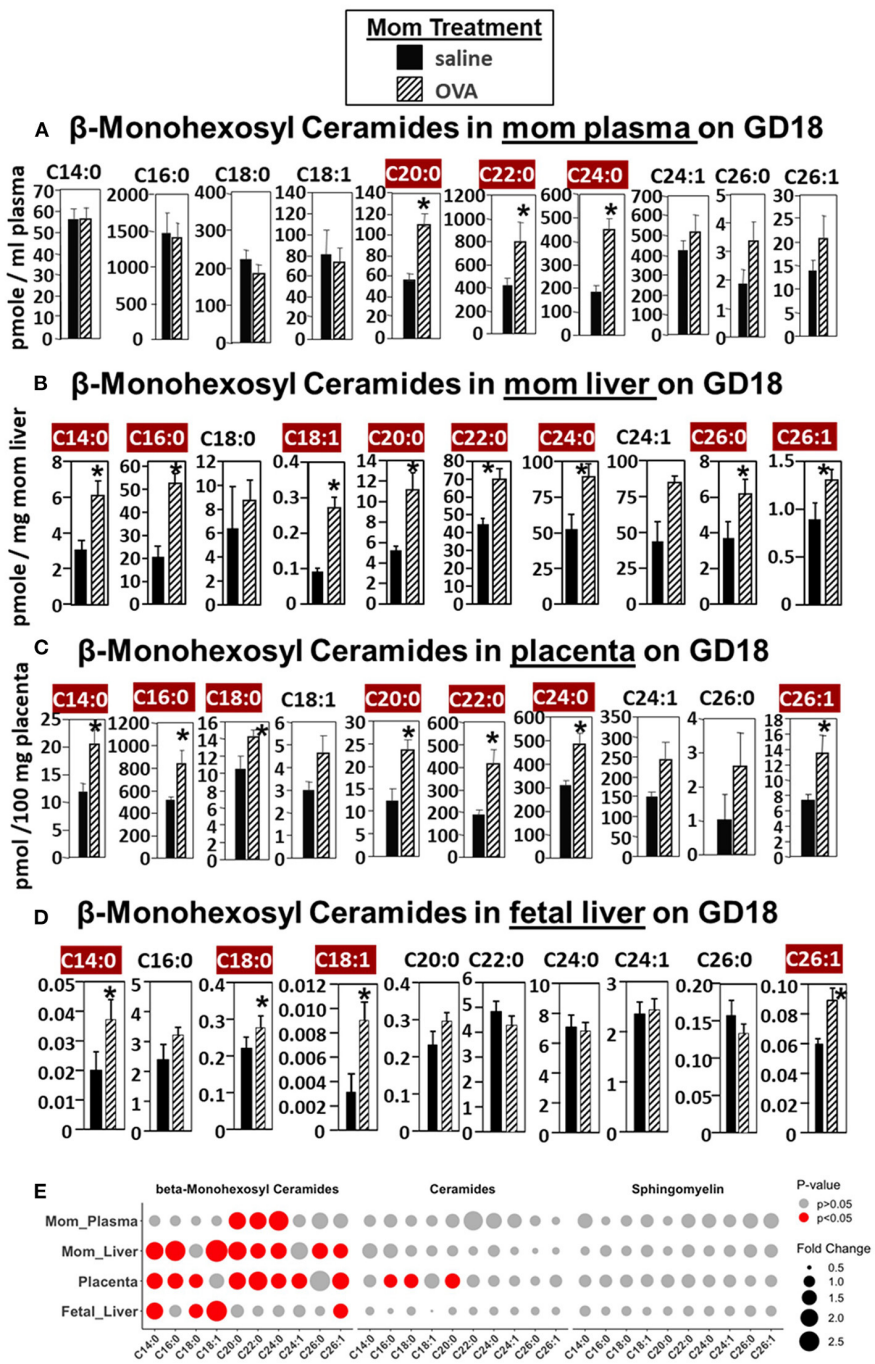
β-monohexosyl ceramides are increased in maternal and fetal tissues of allergic moms. Allergic moms were generated as in the timeline in Figure 1A. Tissue β-monohexosyl ceramides were measured by mass spectrometry in **(A)** mom plasma, **(B)** mom liver, **(C)** placenta, and **(D)** fetal liver on gestational day 18 of OVA-treated and saline-treated mothers. **(E)** Lipidomics bubble plot of fold change and significantly greater in OVA than saline treated groups for β-monohexosyl ceramides, ceramides, and sphingomylins. β-monohexosyl ceramides include both β-glucosyl and β-galactosyl ceramides. Dot size corresponds to fold change between OVA and saline group. *n* = 8–10 mice per group for a representative experiment of two experiments. *p < 0.05 greater in OVA than saline group, crimson red highlighted chain lengths are above the panels.

For allergic mothers, the GD18 maternal plasma, maternal liver, placenta, and fetal liver had elevated βHexCer (Figure 2). The chain lengths that are highlighted in crimson red are for those chain lengths with a significant difference. The plasma from allergic mothers had significantly elevated βHexCer with fatty acid chain lengths of C20:0, C22:0, and C24:0, as highlighted in crimson red in Figure 2A. The mom liver had elevated βHexCer with fatty acid chain lengths of C14:0, C16:0, C18:1, C20:0, C22:0, C24:0, C26:0, and C26:1, as highlighted in crimson red in Figure 2B. Placentas from allergic mothers had elevated βHexCer with fatty acid chain lengths of C14:0, C16:0, C18:0, C20:0, C22:0, C24:0, and C26:1, as highlighted in crimson red in Figure 2C. Fetal liver had elevated βHexCer with fatty acid chain lengths of C14:0, C18:0, C18:1, and C26:1, as highlighted in crimson red in Figure 2D.

In Figure 2E, a summary of lipidomics analysis of βHexCer, ceramides and sphingomyelin indicated that on GD18 the tissues of allergic mothers had elevated βHexCer, had limited increases in placenta ceramides but not fetal liver, and no increases in sphingomyelins. Graphs of data for ceramides and sphingomyelins for this experiment (Supplementary Figures 1, 2) and the rest of the studies in this manuscript are included in Supplementary Figures.

With regards to sphingosines, maternal but not offspring S1P was elevated (Supplementary Figure 3). Briefly, S1P and dihydro-S1P were increased in plasma of allergic mothers on gestational day 18 (GD18) (Supplementary Figure 3A). The placentas on GD18 did not have elevated S1P, dihydro-S1P, or dihydro-sphingosine, but placentas did have elevated sphingosine (Supplementary Figure 3B). This is consistent with the placenta having a 7-fold higher level of S1P phosphatase than most other tissues (50). This restriction of S1P to the placenta is important because S1P is inhibitory for trophoblast development (51). Sphingosine metabolites were not elevated in the fetal liver of allergic mothers (Supplementary Figure 3C). These data indicate that maternal S1P in allergic mothers is metabolized to sphingosine in the placenta and that S1P is not elevated in the fetal liver.

### In the Fetal Liver, the Elevated βHexCer Are Comprised of Increased β-Glucosylceramides (βGlcCer) but Not β-galactosylceramides (βGalCer)

**Figure 3:**
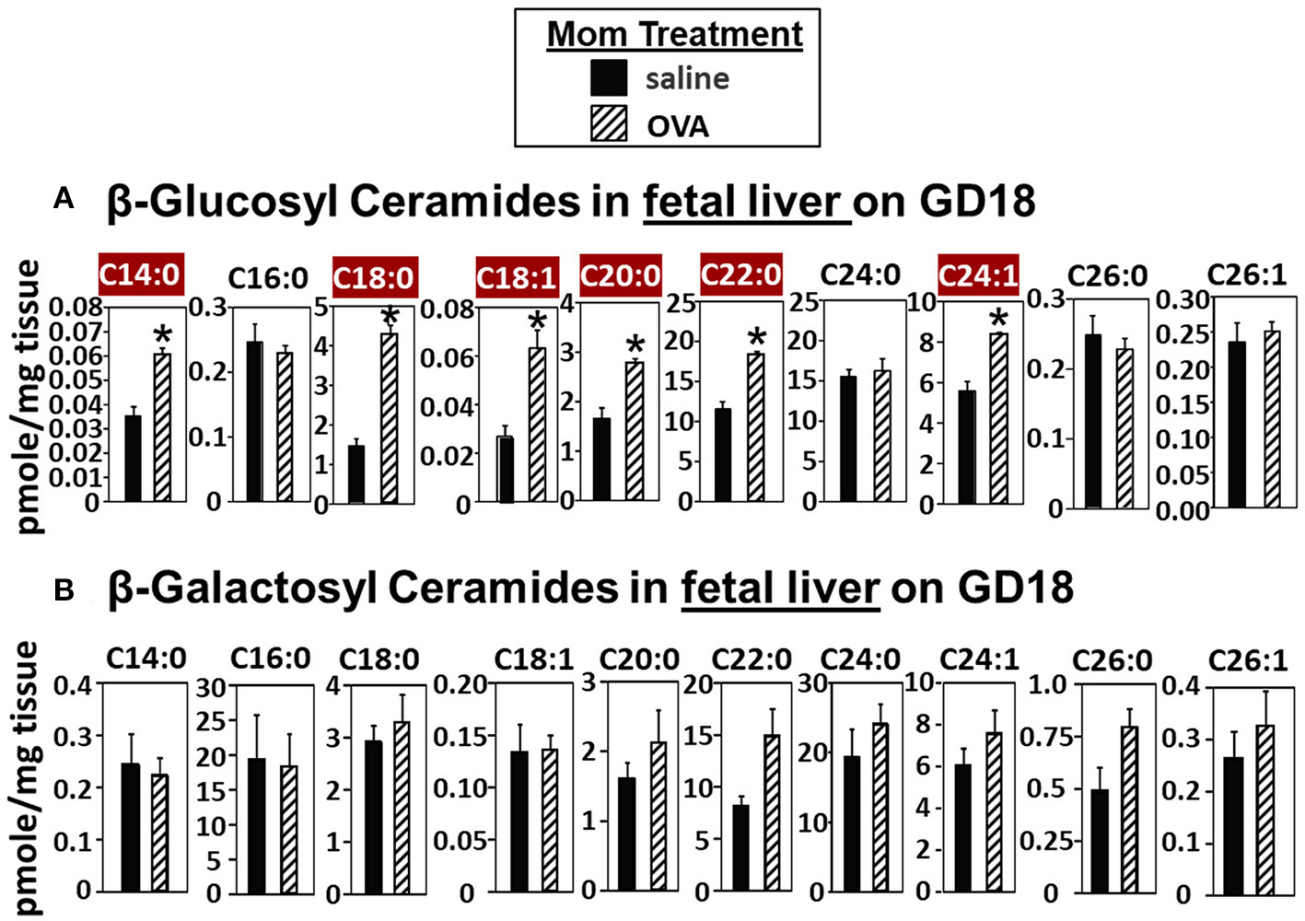
β-glucosyl ceramides are increased in the fetal livers of allergic moms. Mice were treated as in the timeline in Figure 1A. On gestational day 18, fetal liver **(A)** β-glucosyl ceramides and **(B)** β-galactosyl ceramides were separated by column chromatography and then analyzed by mass spectrometry. *n* = 8–10 mice per group for a representative experiment of two experiments. *p < 0.05, crimson red highlighted chain lengths are above the panels.

In Figure 2, there were increases in fetal liver β-HexCer but these analyses did not differentiate between βGlcCer and βGalCer (52). To measure these after lipid extraction of the fetal liver, the βGlcCer and βGalCer were separated by column chromatography and then analyzed by mass spectrometry. In the fetal liver of allergic mothers, there were significant increases in βGlcCer (C14:0, C18:0, C18:1, C20:0, C22:0, and C24:1) but not βGalCer (Figure 3).

### βGlcCer Is Transported Through Maternal Plasma and Maternal Milk to Offspring

**Figure 4:**
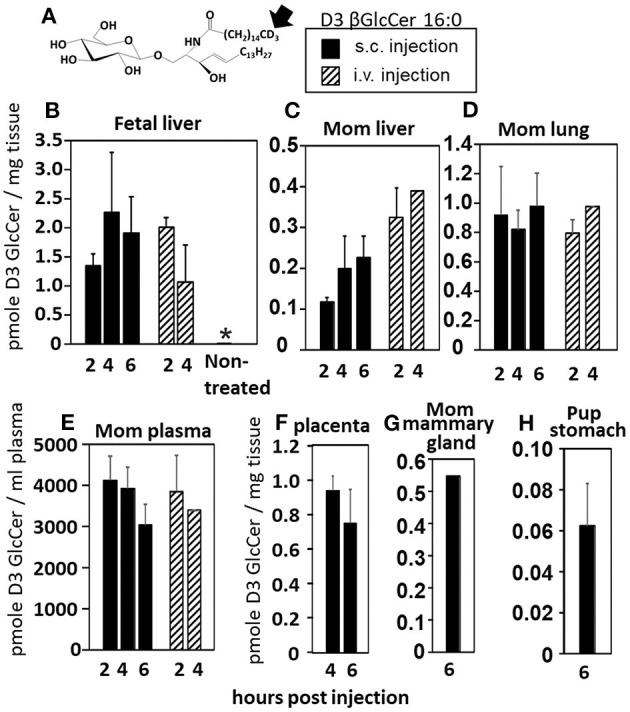
Deuterated C16:0 GlcCer is transported through mom plasma and milk to offspring. **(A)** D3 GlcCer 16:0 (arrow, deuterium position). **(B–F)** On GD18, mothers (timeline Figure 1A) received a subcutaneous injection with D3 GlcCer 16:0 (13 μg/dam) in ethoxylated castor oil or an intraveneous injection with D3 GlcCer 16:0 (18 μg/dam) in autologous plasma. **(G,H)** On PND14, mothers received a subcutaneous injection with D3 GlcCer 16:0 (13 μg/dam) in ethoxylated castor oil. *n* = 3–5 mice per group for a representative experiment of two experiments. *p < 0.05 compared to the other groups.

Increases in βGlcCer in offspring of allergic mothers could occur directly through transport of maternal βGlcCer or indirectly by a maternal mediator that increases βGlcCer in both the mother and offspring. Therefore, it was determined whether maternal βGlcCer is transported through the placenta to the fetus and in maternal milk for pup consumption. In these studies, deuterated (D3) βGlcCer C16:0 (Figure 4A) was administered once on GD18 by either subcutaneous injection or intravenous injection and then maternal and offspring tissues collected 2–6 h later. The fetal liver acquired the D3-βGlcCer within 2 h for both administration routes (Figure 4B). The D3-βGlcCer was also present in mom plasma, mom liver, mom lung, and placenta at GD18 (Figures 4C–F). When D3-βGlcCer was administered by subcutaneous injection to nursing allergic moms at postnatal day 14 (PND14), D3-βGlcCer was present at 6 h in mom mammary glands and pup stomach (Figures 4G,H). These data indicate that maternal βGlcCer is transferred from the mother to the fetuses and pups.

#### Maternal βGlcCer Is Sufficient to Elevate Offspring Lung Eosinophilia in Response to Allergen

**Figure 5:**
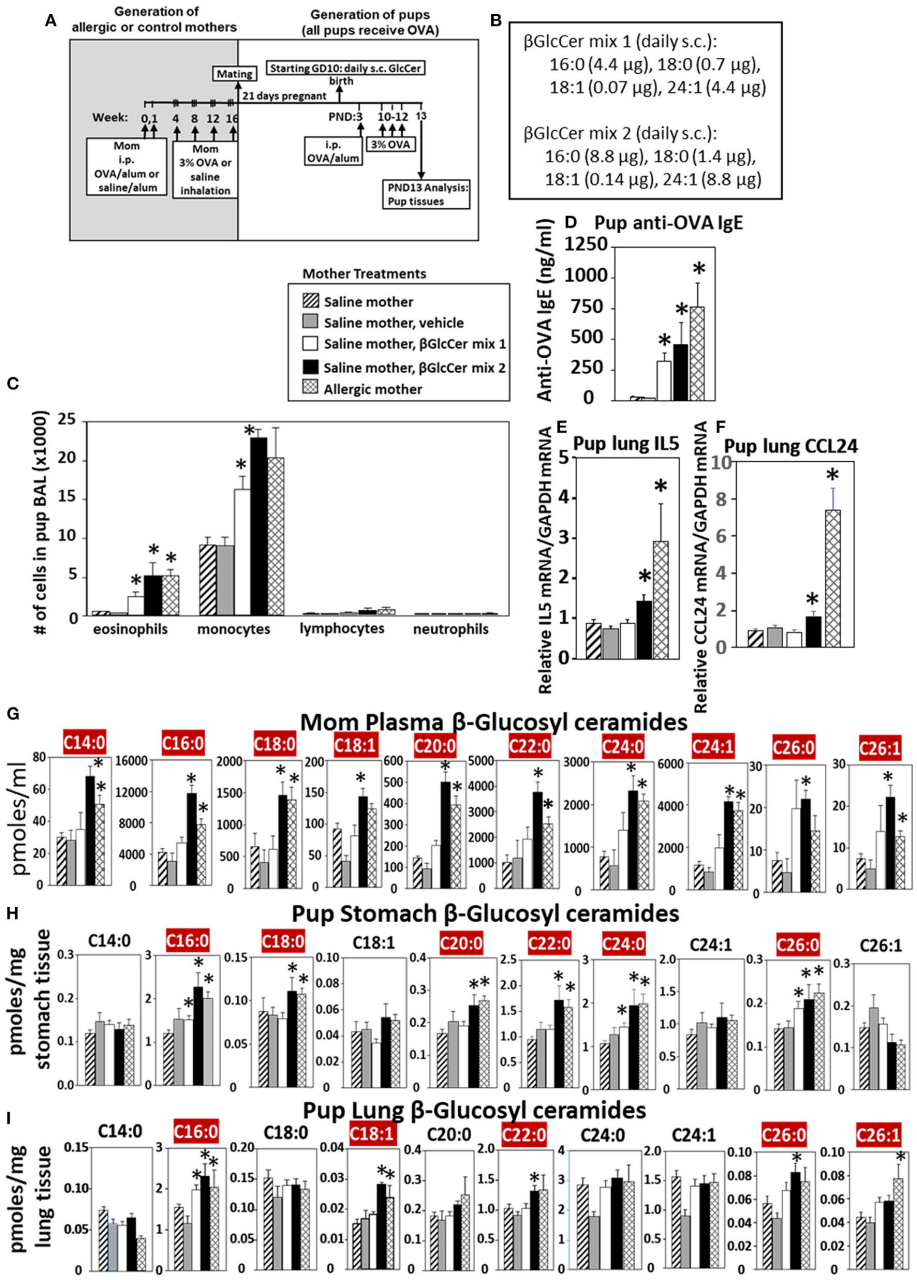
Maternal administration of βGlcCer is sufficient to enhance offspring responses to allergen. **(A)** Mothers were sensitized and challenged with OVA or saline and then mated. Starting on GD10, mothers received daily subcutaneous (s.c.) injections of **(B)** βGlcCer mix 1 or mix 2 composed of βGlcCer 16:0, 18:0, 18:1, and 24:1 at the indicated amounts/pup in vehicle (PEG35, 20% ethanol). At postnatal day 3 (PND3), pups in all groups received one intraperitoneal (i.p) sensitization with OVA/alum. At PND10-12, pups in all groups received 3 challenges with OVA. For experiments with mom and pup analysis, tissues were collected on PND13. **(C)** Bronchoalveolar lavage (BAL) leukocytes of postnatal day 13 OVA-challenged pups from saline (non-allergic) mothers that were non-treated or treated with vehicle or βGlcCer mix 1 or mix 2. Positive controls are pups from allergic mothers. **(D)** Pup serum anti-OVA IgE as determined by ELISA. **(E)** Pup BAL IL5, and **(F)** Pup BAL CCL24 were determined by ELISA. Tissue β-glucosyl ceramides **(G–I)** and β-galactosyl ceramides (Supplementary Figure 4) were separated by column chromatography and then analyzed by mass spectrometry. *n* = 8–10 mice per group for a representative experiment of two experiments. *p < 0.05 compared to saline or vehicle group, crimson red highlighted chain lengths are above the panels.

**Figure 6:**
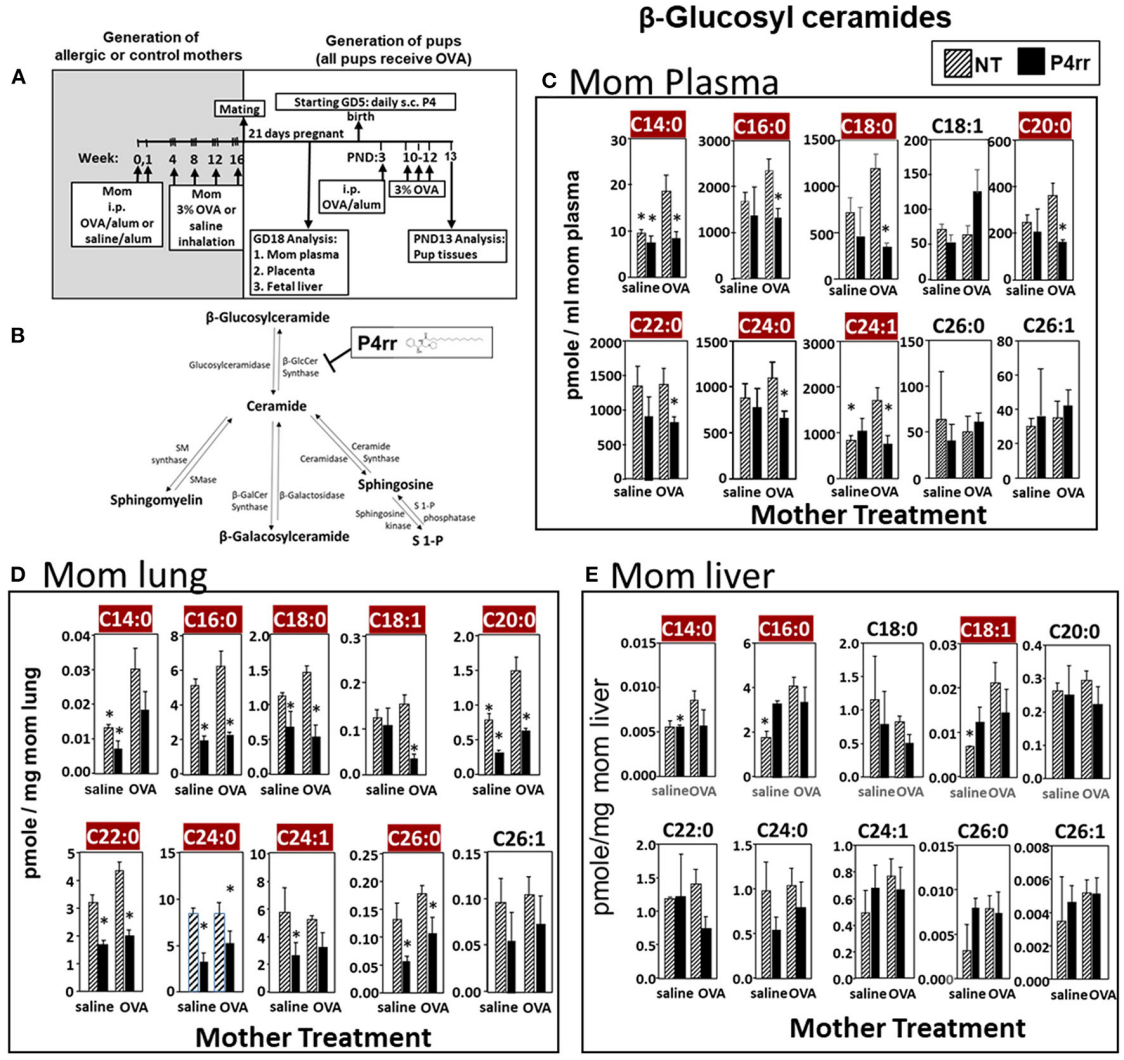
P4rr decreased β-glucosyl ceramides in plasma, lung and liver of allergic mothers. **(A)** Mothers were sensitized and challenged with OVA or saline and then mated. Mothers received daily subcutaneous (s.c.) injections of **(B)** P4rr or the inactive (*S,S*) stereoisomer. P4rr or P4ss were administered during pregnancy (GD5-GD20) and during nursing (PND3-13) to allergic mothers. At postnatal day 3 (PND3), pups in all groups received one intraperitoneal (i.p) sensitization with OVA/alum. At PND10-12, pups in all groups received 3 challenges with OVA. For experiments with maternal and fetal analysis, tissues were collected on GD18. For experiments with pup analysis, tissues were collected on PND13. Tissue β-glucosyl ceramides **(C–E)** and β-galactosyl ceramides (Supplementary Figure 9) were separated by column chromatography and then analyzed by mass spectrometry. **(C)** Mom plasma. **(D)** Mom lung. **(E)** Mom liver. *n* = 8–10 mice per group for a representative experiment of two experiments. *p < 0.05 compared to NT, OVA allergic mother, crimson red highlighted chain lengths are above the panels.

Eosinophilia is a hallmark of allergic lung inflammation. To determine whether maternal βGlcCer is sufficient to elevate offspring lung eosinophilia in response to allergen, non-allergic pregnant dams were administered daily subcutaneous βGlcCer or vehicle starting on GD10 (Figure 5A) because this is near the beginning of development of the immune system and beyond injection-stress-induced early gestational fetal resorption. Additional controls included non-injected non-allergic dams sensitized and challenged with saline and included non-injected OVA-allergic dams (Figure 5A). To determine dose dependent responses, two doses of βGlcCer were administered to dams as a mixture of 16:0, 18:0, 18:1, and 24:1 because these chain lengths are commercially available. The daily dose of 16:0 in mixture 2 was 8.8 μg/dam in Figure 5B because in Figure 4 injection of D3 βGlcCer 16:0 (13 μg/dam) increased mom plasma to 4,100 pmoles/ml, which is about twice that of endogenous allergic mom plasma βGlcCer in Figure 6C. Then, the ratios of 18:0, 18:1, and 24:1 to the 16:0 were set similar to the ratios for concentrations in mom plasma in Figure 6C. The mixture 1 was at half the dose of mixture 2 (Figure 5B). As previously described for enhanced sensitivity of pups of allergic mothers (4–6, 13, 14, 53, 54), the pups received suboptimal allergen sensitization (by one instead of two intraperitoneal OVA/alum injections) and challenged with inhalation of OVA as in Figure 5A. The lung eosinophilia was examined at 24 h after OVA challenge as previously described (5, 12–14) because eosinophilia after OVA challenge increases at 24 h and is sustained up to 9 days with a gradual clearance of lung eosinophilia by 2 weeks (55).

The data demonstrate that there was a dose dependent increase in pup broncho-alveolar lavage eosinophils and monocytes (Figure 5C) and pup serum anti-OVA-specific IgE (Figure 5D) in the pups of βGlcCer-injected non-allergic mother. These levels of inflammation were similar to the level in pups of allergic mothers (Figure 5C). There was also increased pup lung gene expression of the cytokine IL5 and chemokine CCL24 (Figures 5E,F), which are critical for eosinophil development and recruitment, respectively. The administration of βGlcCer to non-allergic mothers elevated βGlcCer in the mom plasma, pup stomach and pup lung (Figures 5G–I), indicating that elevating maternal βGlcCer increases offspring βGlcCer. There was little to no increase in mom or offspring βGalCer (Supplementary Figure 4), ceramides (Supplementary Figure 5), or sphingomyelin (Supplementary Figure 6). Thus, increased maternal βGlcCer was sufficient to enhance offspring lung eosinophilia in response to allergen.

### Inhibition of βGlcCer Synthase Reduced βHexCer of Allergic Mothers to the Levels in Non-allergic Mothers

The data in Figure 5 established that elevation of maternal βGlcCer was sufficient to promote pup lung eosinophilia in response to allergen. It was next determined whether this increase was necessary for the responsiveness to allergen in pups of allergic mothers. To address this, maternal βGlcCer plasma concentrations in allergic mothers were reduced to the level in non-allergic mothers by P4rr, a glucosylceramide transferase inhibitor (56, 57) in a P4 derivative class of βGlcCer inhibitors used in clinical trials as this class has high specificity for βGlcCer and low toxicity (57–61). P4rr was administered by subcutaneous injection daily (57–59, 62) during pregnancy (GD5-GD20) and during nursing (PND3-13) to allergic mothers (Figures 6A,B). The P4rr was started on GD5, which is 6–7 days after the last OVA challenge to the mothers, a time after the OVA-induced 2–5 days peak in eosinophilia and when IL4 and IL5 have resolved in OVA-challenged adult mice (55, 63). The P4rr inhibitor was chosen rather than the D-PDMP inhibitor because P4rr is more selective for βGlcCer transferase than D-PMDP and doesn't have the D-PMDP side effects of increased ceramide storage (60). As controls, a group of allergic mothers received the inactive (S,S) enantiomer, P4ss, at the same dose (59). These doses of P4rr have been shown to reduce plasma βGlcCer by 1/3 to ½ in other models without adverse effects (57–59), and this is the level of reduction needed to bring plasma and fetal liver βGlcCer in allergic mothers to the level of βGlcCer in non-allergic mothers (Figures 2, 3). The pups were suboptimally sensitized [by one instead of two intraperitoneal OVA/alum injections (4–6, 13, 14, 53, 54)] and challenged with inhalation of OVA as in Figure 6A.

The maternal and offspring tissues were examined for βHexCer and βGlcCer. For tissue βHexCer, maternal administration of P4rr, but not the inactive control P4ss, reduced allergic mother plasma βHexCer (C14:0, C16:0, C18:0, C18:1, C20:0, C22:0, C24:0, C24:1, C26:0, and C26:1), allergic mother placenta βHexCer (C14:0, C18:0, C18:1, C20:0, C22:0, C24:0, C24:1, C26:0, and C26:1), and allergic mother fetal liver βHexCer (C14:0, C16:0, C18:0, C18:1, C20:0, and C26:1) (Supplementary Figure 7). In the OVA-challenged pups of allergic mothers, maternal administration of P4rr reduced pup liver βHexCer (C18:0, C18:1, and C20:0) and pup lung βHexCer (C18:1) (Supplementary Figure 8).

**Figure 7:**
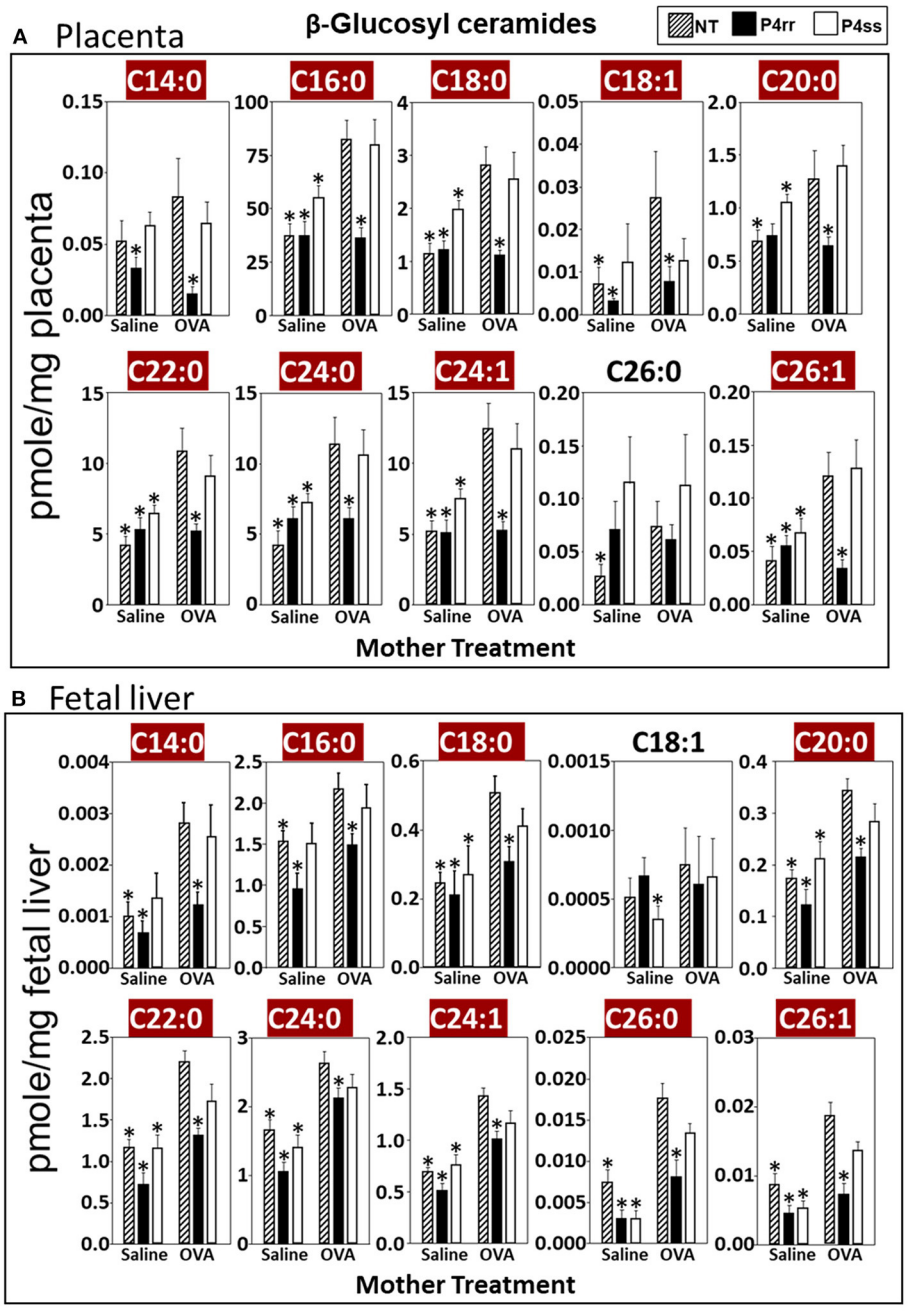
P4rr decreased β-glucosyl ceramides in placenta and fetal liver of allergic mothers. Moms were treated as in Figure 6A and tissues were collected on GD18. Tissue β-glucosyl ceramides **(A,B)** and β-galactosyl ceramides (Supplementary Figure 12) were separated by column chromatography and then analyzed by mass spectrometry. **(A)** Placenta. **(B)** Fetal liver. *n* = 8–10 mice per group for a representative experiment of two experiments. *p < 0.05 compared to NT, OVA allergic mother, crimson red highlighted chain lengths are above the panels.

**Figure 8:**
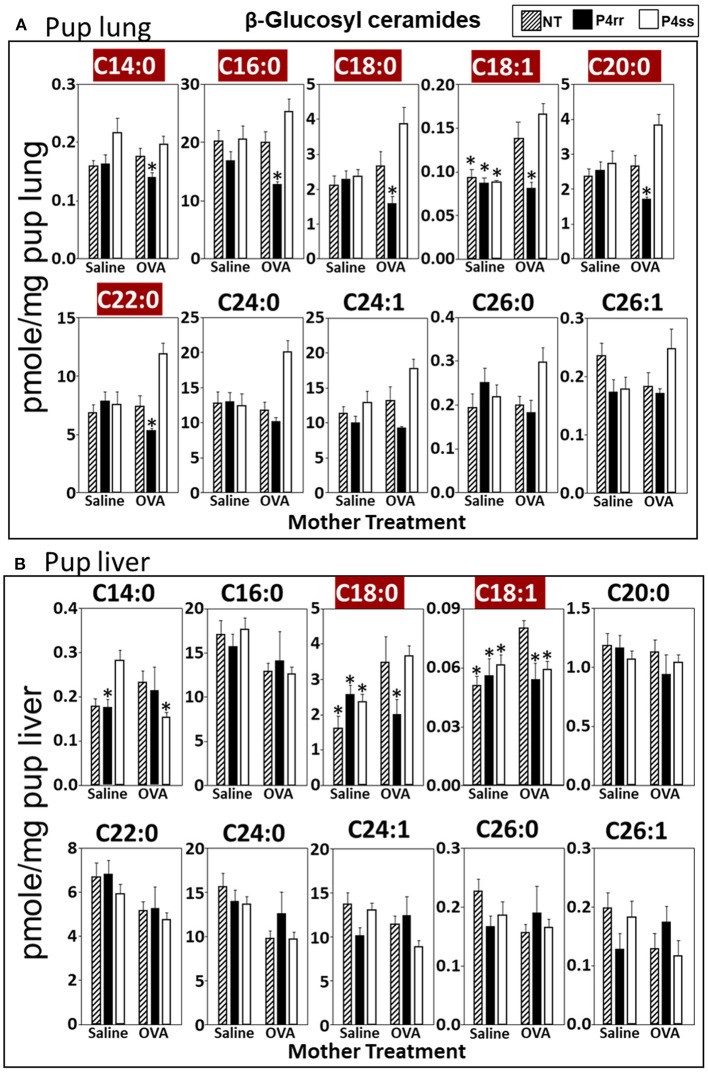
P4rr decreased β-glucosyl ceramides in lung and liver of pups of allergic mothers. Moms were treated as in Figure 6A and pup tissues were collected on PND13. Tissue β-glucosyl ceramides were separated by column chromatography and then analyzed by mass spectrometry. **(A)** Pup lung. **(B)** Pup liver. *n* = 8–10 mice per group for a representative experiment of two experiments. *p < 0.05 compared to NT, OVA allergic mother, crimson red highlighted chain lengths are above the panels.

For tissue βGlcCer, maternal administration of P4rr (Figures 6A,B) reduced the amount of βGlcCer on GD18 in plasma of allergic mothers (C14:0, C16:0, C18:0, C20:0, C22:0, C24:0, and C24:1) (Figure 6C) and lungs of allergic mothers (C14:0, C16:0, C18:0, C18:1, C20:0, C22:0, C24:0, C24:1, and C26:0) (Figure 6D) which is the site of allergic inflammation in the mothers, but there were minimal effects of P4rr on amount of βGlcCer in the liver of allergic mothers (Figure 6E). Placentas of allergic mothers had elevated amounts of βGlcCer (C14:0, C16:0, C18:0, C18:1, C20:0, C22:0, C24:0, C24:1, and C26:1) and this was inhibited by maternal treatment with P4rr but not P4ss (Figure 7A). Fetal livers of allergic mothers had elevated amounts of βGlcCer (C14:0, C16:0, C18:0, C20:0, C22:0, C24:0, C24:1, C26:0, and C26:1) and this was inhibited by maternal treatment with P4rr but not P4ss (Figure 7B). Lungs of allergen-challenged pups from allergic mothers had elevated βGlcCer 18:1 (Figure 8A) and maternal P4rr administration reduced pup lung βGlcCer (C18:0, C18:1, C20:0, and C22:0) (Figure 8A). Allergen-challenged pups of allergic mothers had increased liver βGlcCer (C18:0 and C18:1) which was reduced by maternal P4rr administration (Figure 8B). P4rr did not alter βGalCer in allergic mother plasma, lung or liver (Supplementary Figure 9).

Importantly, P4rr is an inhibitor of βGlcCer synthase and doesn't affect storage of the other lipid metabolites (60). Thus, it was necessary to determine whether there were effects of P4rr on βGalCer, ceramides, sphingomyelins or sphinogosines in this model. Maternal administration of P4rr did not reduce fetal liver sphingosine metabolites (Supplementary Figure 3D). With regards to ceramides, maternal P4rr did not reduce allergic mom plasma ceramides (Supplementary Figure 10A), allergic mom liver ceramides (Supplementary Figure 10C), or the majority of allergic mom lung ceramides except the allergic mom lung ceramides (C14:0, C18:0, C18:1) (Supplementary Figure 10B). With regards to sphingomyelins, maternal P4rr did not reduce sphingomyelins in plasma (Supplementary Figure 11A) or lungs (Supplementary Figure 11B) of allergic moms; also, there was no reduction in the majority of sphingomyelins in allergic mom liver, except the allergic mom liver sphingomyelins C18:0 and C18:1 (Supplemental Figure 11C).

In placentas of allergic mothers, βGalCer was elevated and this was reduced by maternal P4rr, but not maternal P4ss (C14:0, C16:0, C18:0, C18:1, C20:0, C22:0, C24:0, C24:1, C26:0, and C26:1) (Supplementary Figure 12A). Importantly, in contrast to placentas, fetal liver βGalCer was not elevated and P4rr did not reduce fetal liver βGalCer in allergic mothers (Supplementary Figure 12B). For offspring of allergic mothers, maternal P4rr did not reduce ceramides in placentas (Supplementary Figure 13A), fetal livers (Supplementary Figure 13B), and pup lungs (Supplementary Figure 13C), but reduced only ceramide C18:0 in pup liver (Supplementary Figure 13D). In offspring of allergic mothers, maternal P4rr had minimal reduction in sphingomyelin (C16:0) in placentas (Supplementary Figure 14A) and did not alter sphingomyelins levels in fetal liver (Supplementary Figure 14B), pup lung (Supplementary Figure 14C), and pup liver (Supplementary Figure 14D).

P4rr is reported to inhibit activity of βGlcCer synthase. It was also determined whether expression of enzymes of the ceramide pathway were not altered. There were no effects of allergen or P4rr on expression of enzymes for *de novo* synthesis of ceramide (DEGS gene encodes dihydroceramide desaturase), expression of βGlcCer synthase (UGCG), or enzymes for degradation of βGlcCer to ceramide (GBA gene encodes β-glucosidase) (Supplementary Figure 15). Thus, the βGlcCer synthase inhibitor P4rr reduced maternal βGlcCer without altering the ceramide pathway enzyme gene expression and without global effects on fetal or pup levels of βGalCer, ceramides, or sphingomyelins.

### Inhibition of βGlcCer Synthase Blocks Allergen-Induced Lung Eosinophilia in Offspring of Allergic Mothers

**Figure 9:**
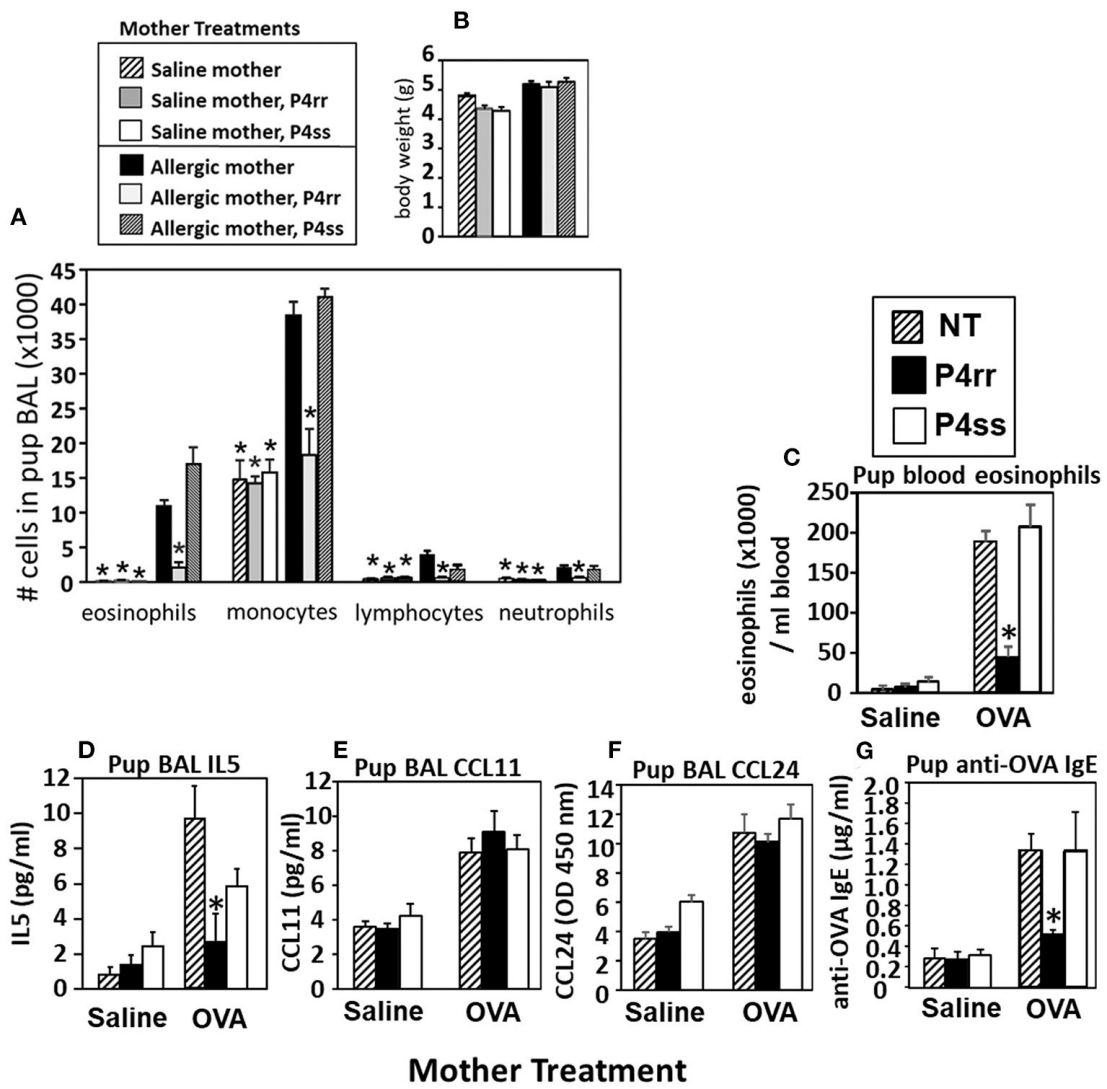
P4rr decreased lung lavage inflammation and inflammatory mediators in OVA-challenged pups from allergic mothers. **(A)** Bronchoalveolar lavage (BAL) leukocytes of postnatal day 13 OVA-challenged pups from allergic and saline (non-allergic) control mothers that were non-treated (NT) or treated with P4rr or P4ss. **(B)** Body weight. **(C)** Pup blood eosinophil numbers. **(D)** Pup BAL IL5, **(E)** Pup BAL CCL11, **(F)** Pup BAL CCL24 as determined by ELISA. **(G)** Pup serum anti-OVA IgE as determined by ELISA. *n* = 8–10 mice per group for a representative experiment of two experiments. *p < 0.05 compared to the pups of the OVA-treated moms and pups of the OVA P4ss treated moms.

The pups from Figure 6A, were examined for allergen-induced lung eosinophilia. There were no effects of P4rr, P4ss, or allergen treatments on pup body weight (Figure 9B), pup numbers, or gender distribution (data not shown). In allergen-challenged offspring of allergic mothers, maternal administration of P4rr, but not the inactive enantiomer P4ss, blocked the allergen-induced increases in bronchoalveolar lavage eosinophils and monocytes and the low levels of lymphocytes and neutrophils (Figure 9A). In allergen-challenged offspring of allergic mothers, maternal administration of P4rr blocked the increase in pup blood eosinophils (Figure 9C) and blocked the increase in pup lung IL-5 (Figure 9D), which mediates bone marrow differentiation and recruitment of eosinophils to the lung in allergic responses (64). Pups of allergic mothers had elevated CCL11 and CCL24 but this was not affected by P4rr (Figures 9E,F). The offspring of non-allergic mothers did not respond to allergen challenge and the P4rr did not alter the low levels of leukocytes in these offspring of non-allergic mothers (Figure 9A). Also, P4rr, but not the inactive P4ss control, blocked the allergen-induced increased in anti-OVA specific IgE in pups of allergic mothers (Figure 9G).

**Figure 10:**
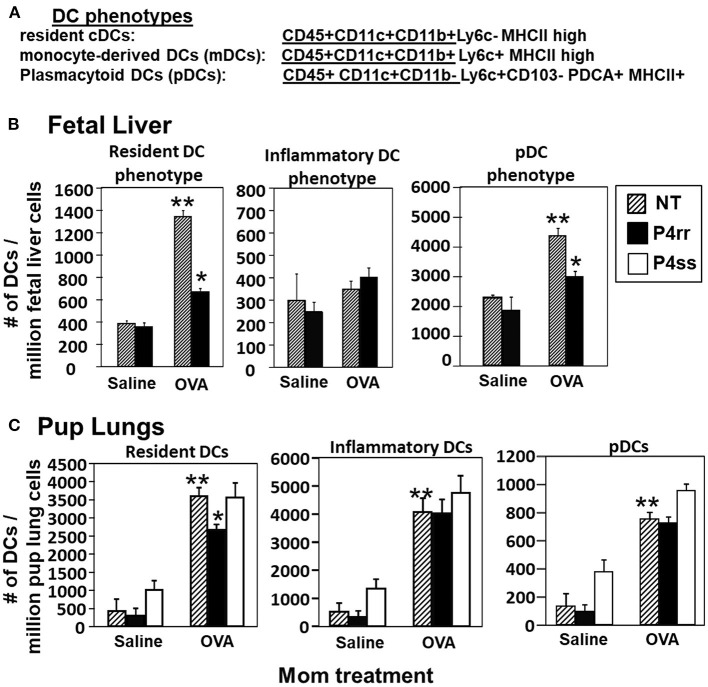
P4rr treatment of allergic mothers inhibited the increase in numbers of dendritic cells in the fetal livers and lungs of pups of allergic mothers. The fetal livers or the pup lung tissues were collected from mice treated as in Figure 6A. Tissue dendritic cells were immunolabeled and examined by flow cytometry. **(A)** Chart of phenotype of lung CD11c+ subsets analyzed in the fetal livers and pup lungs. **(B)** Numbers of CD11c+ DC subsets per millibon fetal liver cells. **(C)** Numbers of CD11c+ DC subsets per million pup lung cells. *n* = 8–10 mice per group for a representative experiment of two experiments. **p < 0.05 compared to pups of NT, saline mother. *p < 0.05 compared to pups of NT, OVA allergic mother.

The fetal livers and lungs of allergen-challenged pups of allergic mothers have elevated numbers of resident DCs (13, 14). Therefore, it was determined whether this increase in DCs was blocked by P4rr as determined by immunolabeling (13, 14) (Figure 10A) and flow cytometry. The fetal livers of allergic mothers had elevated numbers of DCs with the phenotype of resident DCs and plasmacytoid DCs (pDCs) and this increase in DCs was blocked by maternal P4rr (Figure 10B). Lungs of allergen-challenged pups of allergic mothers had increased numbers of resident DCs, inflammatory DCs and pDCs (Figure 10C). Maternal administration of P4rr reduced the numbers of the resident DCs in pup lungs (Figure 10C). Thus, P4rr reduced fetal liver βGlcCer, pup lung βGlcCer, pup liver βGlcCer, OVA-specific IgE, and the pup allergen-induced allergic inflammation, consistent with the P4rr-mediated reduced numbers of resident DCs in the fetal liver and pup lungs and demonstrating that βGlcCer was necessary for the increased allergen-induced lung eosinophilia in offspring of allergic mothers.

## Discussion

**Figure 11:**
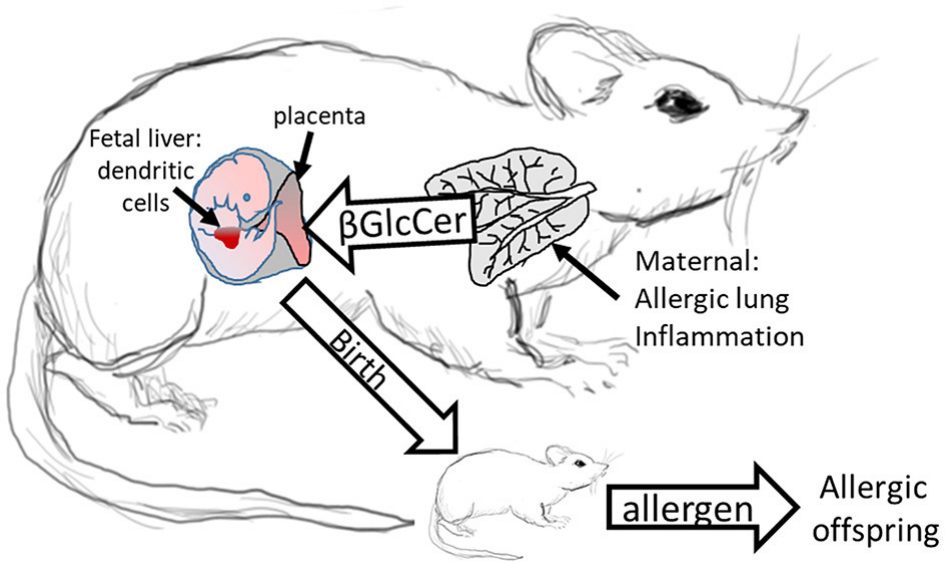
Model for Maternal βGlcCer enhancement of offspring allergen responsiveness. Mothers with allergic lung inflammation have increased βGlcCer that is transported to the placenta and the fetal liver, where the immune system develops in the fetus. This increases DCs of resident DC phenotype in the fetal liver and neonates have elevated numbers of resident DCs in the pup lungs. These neonates of allergic mothers develop allergic lung inflammation in response to allergen sensitization and allergen challenge. The neonate response to allergen is mediated by altered dendritic cell development since DCs of offspring of allergic mothers are sufficient for the allergen responsiveness (4, 12). In contrast, offspring of non-allergic mothers do not have elevated βGlcCer and don't respond to allergen.

We demonstrated that in allergic mothers, increased maternal βGlcCer is necessary and sufficient to increase offspring lung eosinophilia in response to allergen. In allergic mothers on GD18, there was an increase in several isoforms of βGlcCer in mom plasma and mom lung. The increased maternal plasma βGlcCer was transported to the fetus, fetal liver, and breast tissue, and was in the pup stomach. Increasing maternal βGlcCer of non-allergic moms was sufficient for elevated offspring lung eosinophilia in response to allergen. Furthermore, the increase in fetal liver βGlcCer elevated numbers of fetal liver resident phenotype of DCs because the increase in βGlcCer in allergic mothers and their offspring was inhibited by maternal administration of a clinically relevant inhibitor of βGlcCer synthase, P4rr, during pregnancy, and nursing. The P4rr had high specificity for βGlcCer because it significantly reduced βGlcCer but had little to no effect on ceramides, sphingomyelins, or sphingosines. P4rr did not alter βGalCer in mom lung, mom plasma, mom liver, fetal liver or pup stomach but had some inhibitory effect on placental βGalCer. P4rr also did not alter mom lung gene expression of the ceramide pathway enzymes. Importantly, the maternal administration of the βGlcCer synthase inhibitor blocked allergen induced lung eosinophilia in the offspring of allergic mothers, consistent with the reduction of βGlcCer in allergic mothers and their offspring to levels of βGlcCer in the non-allergic groups. There was no effect of an inactive stereoisomer of the same molecule, P4ss, on levels of βGlcCer and there was no effect of P4ss on allergen responsiveness in offspring of allergic mothers. These data indicate that elevated βGlcCer in allergic mothers function as a maternal factor that is transported to offspring *in utero* and during nursing. This then elevates offspring development of DCs and allergen induced offspring lung eosinophilia (Figure 11).

The lipids βHexCer and S1P are reported to have pro-inflammatory properties in allergy (20, 21) and in other models of inflammation (20, 21, 37, 38). Reports indicate that in adults, S1P is elevated during allergic responses (20), is transported on HDL/LDL (33, 34), and regulates dendritic cell function (20, 35). However, despite S1P being increased in plasma of allergic mothers, it was not increased in the placenta or fetal liver of allergic mothers, which suggests that the fetus is protected from increases in maternal S1P during development. The increase in a metabolite of S1P, sphingosine, in the placenta is consistent with the high levels of sphingosine-1-phosphate phosphatase in the placenta as compared to other tissues (50).

During offspring development, maternal lipids are transported on LDL and HDL particles that cross the placenta to the fetus (19) and are in the mother's milk during nursing (30–32). LDL and HDL bind the lipid metabolite βGlcCer (65, 66). Lipid metabolites regulate DC differentiation/function (26–29), and DCs mediate initiation of allergic disease (4, 12). It is reported that βGalCer is required for hematopoiesis (67) and that ceramide is elevated in allergic non-pregnant female mice (68). In our studies, βGalCer and ceramide was present but not elevated in the allergic mothers and their offspring. In another disease, graft vs. host disease, administration of βGlcCer enhances IL-4 (38) and in the graft vs. host disease, the immunomodulatory effects of βGlcCer are dependent upon DCs (38). In contrast, one report suggests that allergic inflammation can be reduced by very high doses of a βGlcCer administered 14 times to the lung, using βGlcCer of unreported chain lengths and source (41). In our studies, the endogenous synthesis of βGlcCer in allergic mothers elevated fetal liver DCs, pup lung DC subsets and pup lung eosinophilia. The increase in numbers of fetal liver DCs of the resident phenotype was mediated by βGlcCer from allergic mothers because the increase in these DCs was blocked upon inhibiting the increase in βGlcCer by maternal administration of the βGlcCer synthase inhibitor. Of note, mammalian HexCer are β enantiomers. In contrast, α enantiomers of HexCer are synthesized by bacteria and bind to CD1d on dendritic cells (69). The regulatory functions of maternal βGlcCer on development of offspring DCs and neonate allergic responses are novel. The ligands for βGlcCer during regulation of differentiation of DCs in the fetal liver are unknown and are currently under investigation, although a manuscript is in preparation describing βGlcCer regulation of dendritic cell differentiation *in vitro*.

It is reported that the adoptive transfer of splenic dendritic cells (but not macrophages) from neonates of allergic mothers to recipient neonates from non-allergic mothers transfers allergic responsiveness to the recipient neonates (4, 12). This indicates that DCs are sufficient for this transfer of allergen responsiveness. We have demonstrated that the fetal liver and offspring of allergic mice have increased numbers of distinct CD11b+ subsets of CD11c+ DCs (13, 14). In this report, the increase in CD11b+ subsets of CD11c+ DCs was blocked by treatment of the allergic mothers with a βGlcCer synthase inhibitor. Thus, maternal βGlcCer elevates offspring DCs and allergen responsiveness. This is consistent with reports that offspring of allergic mothers have increased responsiveness to allergen (4–11) and consistent with most reports indicating that offspring allergy is associated with maternal asthma rather than paternal asthma (4). It is acknowledged that other maternal factors may function in concert with βGlcCer for regulation of offspring responsiveness to allergen. We are currently investigating other maternal lipids, especially decreases in anti-inflammatory lipids in allergic mothers.

Mediators of allergic disease include allergen-specific IgE, chemokines that recruit eosinophils and IL-5 that induces eosinopoiesis. OVA-specific IgE and IL-5 were increased in allergen-challenged offspring of allergic mothers but not in non-allergic mothers, and both the IgE and IL-5 were significantly reduced by the βGlcCer synthase inhibitor, consistent with regulation of DCs that are critical for the initiation of allergic responses. The increase in eosinophils in the blood of neonates of allergic mothers and the regulation by βGlcCer synthase inhibitors, suggests that βGlcCer regulates signals for production of IL-5 from immune cells that then induces bone marrow differentiation of eosinophils, resulting in the increased numbers of eosinophils in the lungs of the neonates of allergic mothers. In contrast, there was minimal effect of βGlcCer on CCL24 mRNA expression in the lung tissue because there was only a 1.5 fold increase in lung CCL24 mRNA in pups of non-allergic mothers administered βGlcCer as compared to a large 7 fold increase in CCL24 in lung BAL of pups of allergic mothers. Furthermore, levels of CCL11 and CCL24 protein in pup lung BAL were not regulated by the βGlcCer synthase inhibitor. These data suggest that the production of CCL24 by epithelial cells in response to OVA exposure is induced by a pathway largely independent of βGlcCer.

Interestingly, at 18 days after the last allergen challenge of the mothers, βGlcCer was increased in the plasma of allergic mothers and fetal livers. Moreover, increased βGlcCer was present in the offspring of allergic mothers at PND14 which was 5 weeks after the last allergen challenge of the mother. Furthermore, the maternal βGlcCer was transported to the breast tissue and was in the milk in stomach of nursing pups. βGlcCer is also present in human milk (70), and further studies are in progress to examine association of βGlcCer with allergic disease in humans. In comparing human ages with pup age for the treatments in this report, at PND3 the pups are similar to infants, at PND13 the pups are similar to early childhood (71, 72), which is a stage at which infants and children are exposed to allergens on the skin and can develop allergy to foods. The levels of βGlcCer in human plasma of offspring of allergic mothers is in a manuscript in preparation.

It is important to emphasize that, in these studies, all pups of allergic mothers and all pups of non-allergic mothers received the allergen but only pups of allergic mothers had elevated βGlcCer and developed allergic responses, indicating the specificity of this response in offspring of allergic mothers. The increase in βGlcCer in allergic mothers was lipid specific, because in the fetus and neonates of allergic mothers, there was not an increase in βGalCer, ceramides, or sphingomyelins. The total amount of each isoform of βGlcCer varied, but the abundance of isoforms likely equates to specific functions of the isoforms in membranes and cell signaling; this is currently under investigation. The isoforms of βGlcCer that were most often elevated in mothers and offspring were the short to medium chain length isoforms of βGlcCer. The experiments with transport of deuterated βGlcCer demonstrates that βGlcCer is readily transported to the fetal liver. The P4rr-induced reduction in levels of βGlcCer in fetal livers of allergic mothers to the level in fetal livers of non-allergic mothers resulted in inhibition of the development of allergy in the offspring and mitigated the increase in DCs in the fetal liver. The βGlcCer isoforms have different concentrations in the tissues but concentration does not reflect potency; studies addressing functional concentrations of the isoforms *in vitro* and *in vivo* are ongoing and beyond the scope of this manuscript.

In summary, our innovative studies on allergic inflammation provide evidence that endogenously synthesized maternal lipid metabolites of allergic mothers have regulatory functions in their offspring. The allergic mothers had elevated lung and plasma βGlcCer that were transferred across the placenta to the fetal liver and in milk to the neonate. This increase in βGlcCer mediated an increase in fetal liver resident phenotype DCs and neonate lung DCs as well as allergen responsiveness in the neonates. In conclusion, our studies identify βGlcCer as a maternal factor that is induced in the mother by environmental allergen exposure. This is a critical novel insight into the understanding of the complex interactions of environmental factors and genetic factors in allergy and asthma (73). Whether the regulation by βGlcCer extends beyond allergy should be addressed in future studies. The identification of βGlcCer as a maternal factor from allergic mothers and identification of mechanisms that regulate development of allergy and asthma in offspring of allergic mothers significantly advance this field and may have impact on potential novel approaches for prevention or intervention in asthma and allergies.

## Capsule Summary

β-glucosylceramide of allergic mothers is necessary and sufficient to increase allergen-induced lung eosinophilia in offspring of allergic mothers.

## Data Availability Statement

The original contributions presented in the study are included in the article/Supplementary Material, further inquiries can be directed to the corresponding author/s.

## Ethics Statement

The animal study was reviewed and approved by Indiana University Institutional Review Committee for animals, Indiana University School of Medicine, Indianapolis, IN.

## Author Contributions

MW and RF participated in performing and interpreting experiments. AH performed statistical analysis of lipidomics. LS-C performed qPCR and figure preparations. JC participated in figure and manuscript preparation. RS provided P4rr and P4ss, participated in discussions, interpretations, and manuscript preparation. JC-M conceived of the study design and participated in performing experiments, statistical analysis, interpretations, and manuscript preparation. All authors contributed to the article and approved the submitted version.

## Conflict of Interest

The authors declare that the research was conducted in the absence of any commercial or financial relationships that could be construed as a potential conflict of interest. The handling editor declared a shared affiliation with one of the author RS at time of review.

## Abbreviations

βGlcCer: β-monoglucosylceramide
βGalCer: β-monogalactosylceramide.
