# Supplementary material for: β-Glucosylceramide From Allergic Mothers Enhances Offspring Responsiveness to Allergen
Source: Front Allergy. Author manuscript; Available in PMC 2021 Aug 6. (PMC8345025; doi:10.3389/falgy.2021.647134)
Supplement: Supplementary file 1 [file NIHMS1719304-supplement-1.pdf]

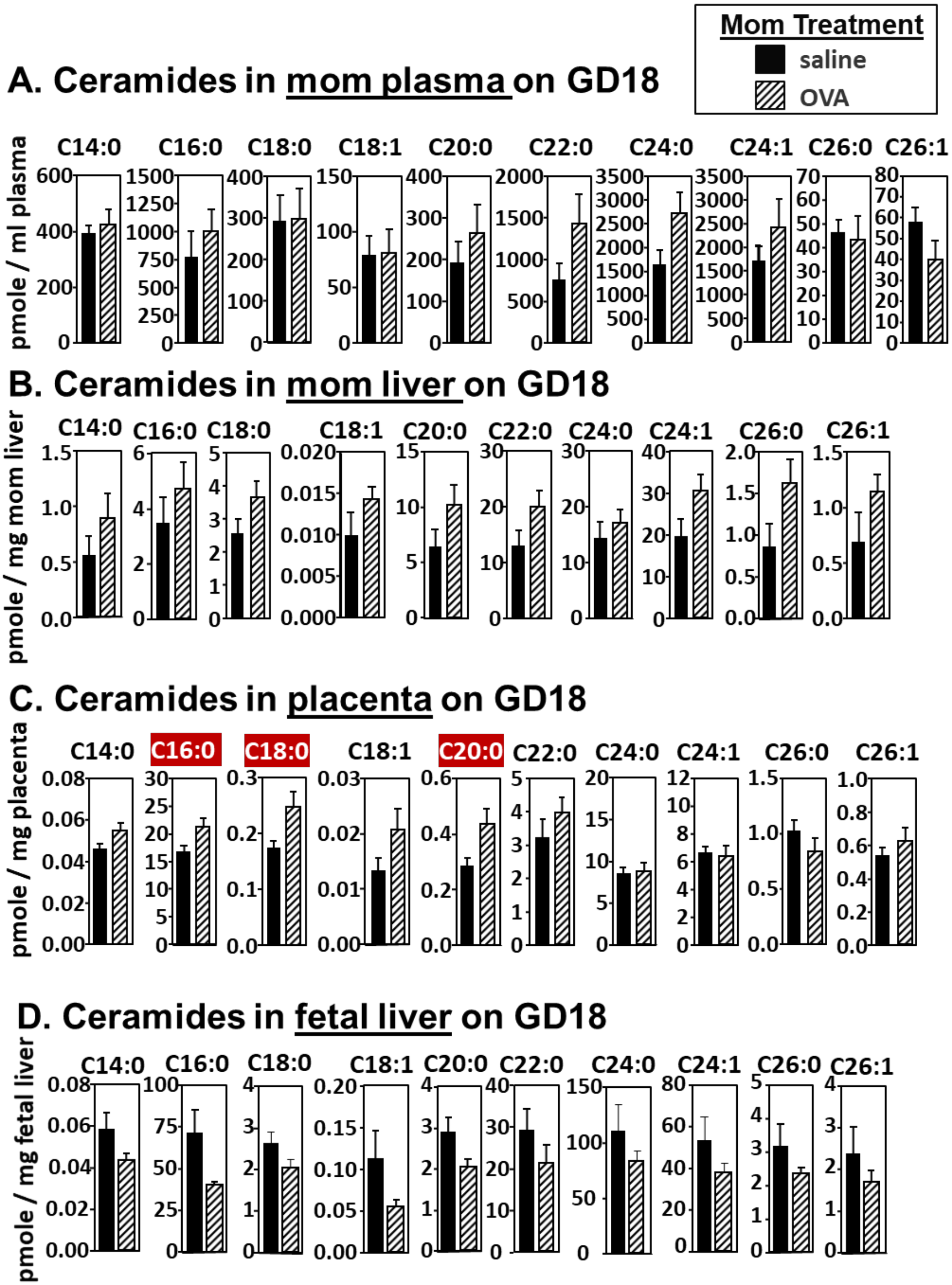

Supplement Figure 1. Ceramides were not altered in maternal and fetal tissues of allergic moms. The mice were treated as in the timeline in main manuscript Figure 1A. Ceramides were measured by mass spectrometry in **A)** mom plasma, **B)** mom liver, **C)** placenta, and **D)** fetal liver on gestational day 18 of OVA-treated and saline-treated mothers from main manuscript Figure 2. n=8-10 mice per group for a representative experiment of two experiments. $p<0.05$ greater in OVA than saline group; crimson red highlighted chain lengths are above the panels.

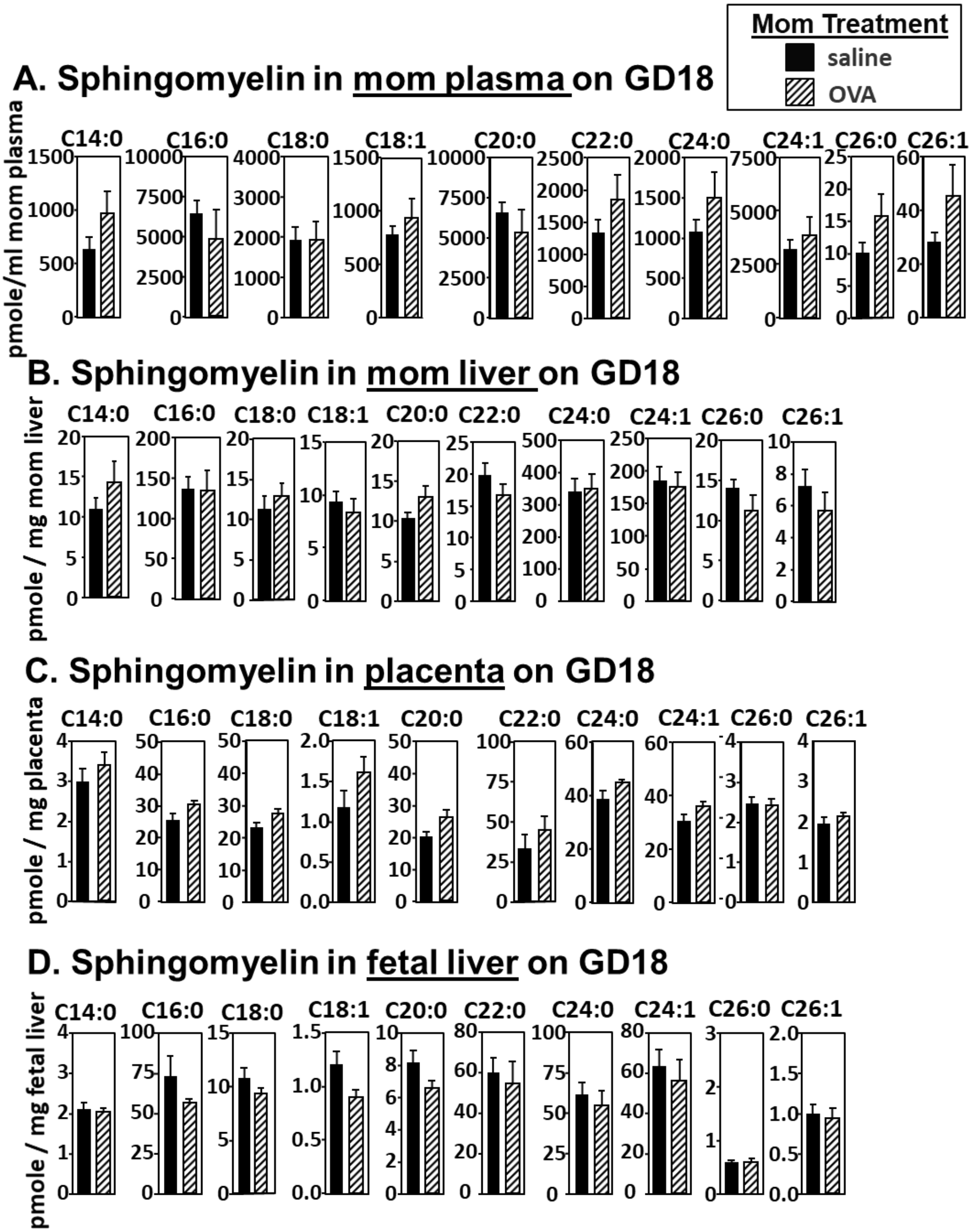

Supplement Figure 2. Sphingomyelins were not altered in maternal and fetal tissues of allergic moms. Mice were treated as in the timeline in main manuscript Figure 1A. Sphingomyelins were measured by mass spectrometry in **A)** mom plasma, **B)** mom liver, **C)** placenta, and **D)** fetal liver on gestational day 18 of OVA-treated and saline-treated mothers from main manuscript Figure 2. n=8-10 mice per group for a representative experiment of two experiments.

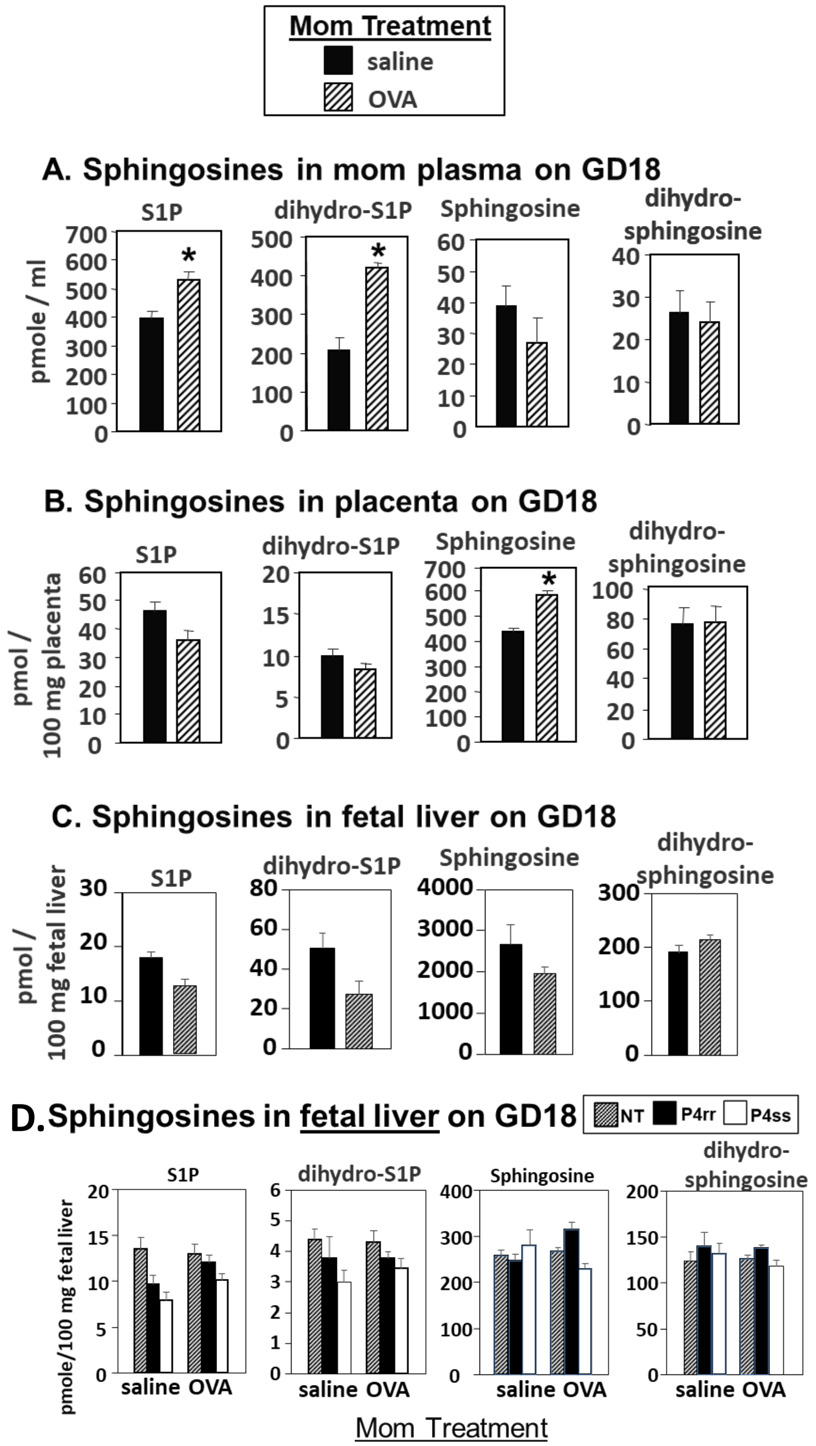

Supplement Figure 3. Sphingosines were increased in mom plasma and placenta but not fetal liver of allergic moms. Also P4rr did not alter sphingosines in the fetal livers of allergic mothers. A-C) Mice were treated as in the timeline in main manuscript Figure 1A. Sphingosines were measured by mass spectrometry in **A)** mom plasma, **B)** placenta, and **C)** fetal liver on gestational day 18 of OVA-treated and saline-treated mothers from main manuscript Figure 2. **D)** Moms were treated as in Figure 4, fetal liver was collected on GD18, and sphingosines were determined by mass spectrometry. n=8-10 mice per group for a representative experiment of two experiments.n=8-10 mice per group for a representative experiment of two experiments. *, $p<0.05$.

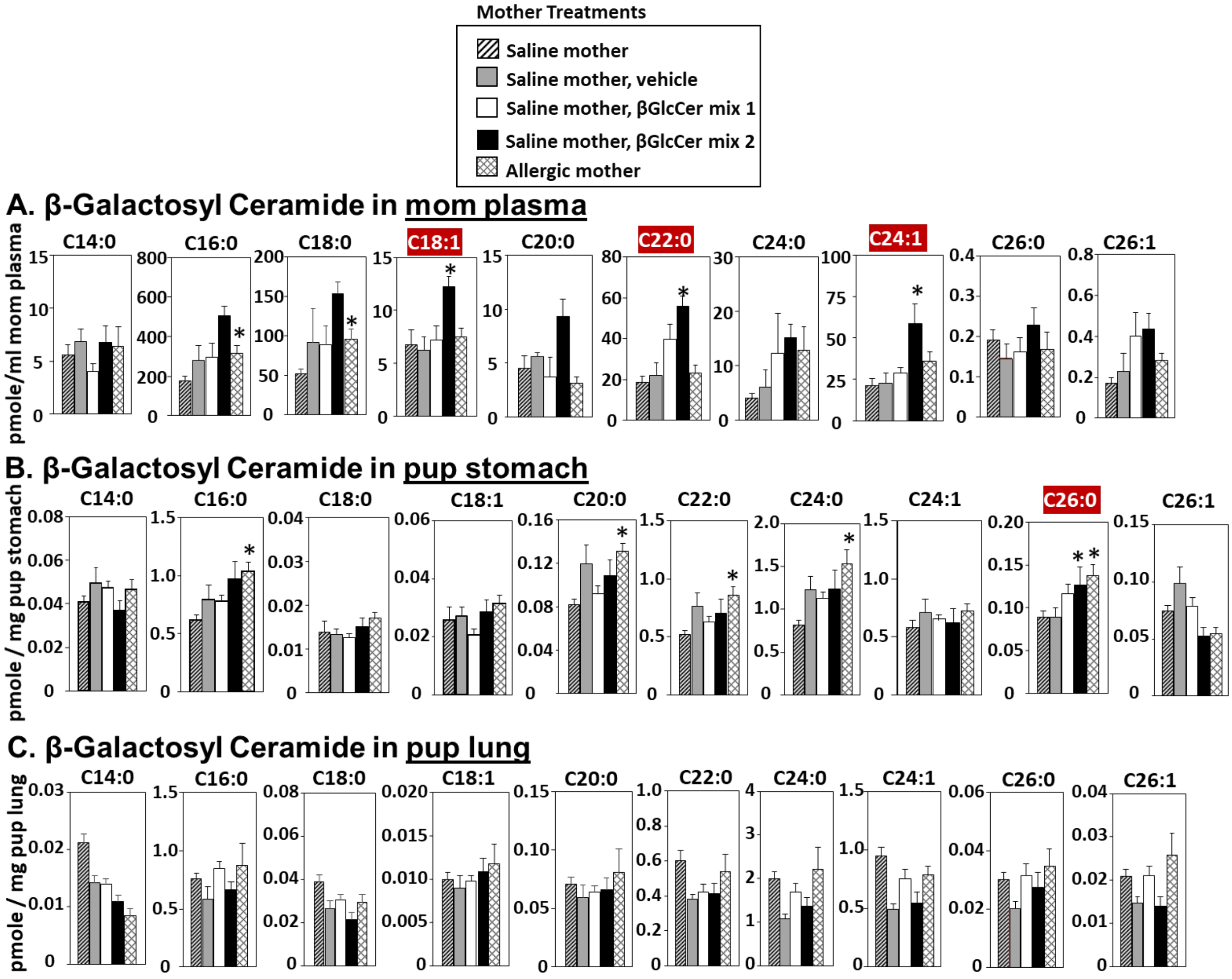

Supplement Figure 4. Maternal administration of βGlcCer had little to no effect on mom and pup βGlcCer. Tissues were from mothers and offspring in Figure 5, where mothers received daily subcutaneous (s.c.) injections of βGlcCer mix 1 or mix 2. Tissue β-galactosyl ceramides from **A)** mom plasma, **B)** pup stomach and **C)** pup lung were separated by column chromatography and then analyzed by mass spectrometry. n=8-10 mice per group for a representative experiment of two experiments. *, $p<0.05$ compared to saline or vehicle group; for significant differences of βGlcCer mix groups versus vehicle groups the crimson red highlighted chain lengths are above the panels.

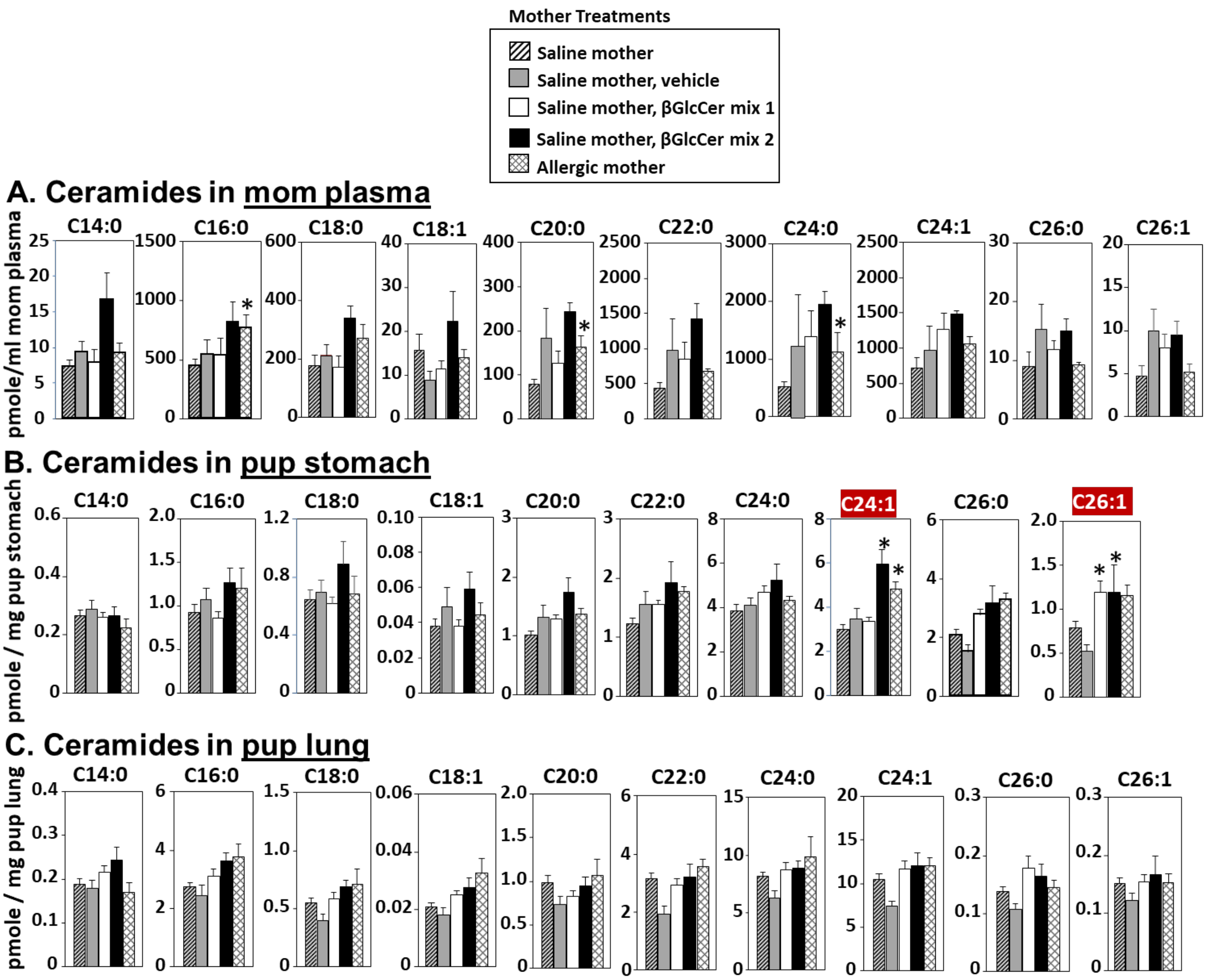

Supplement Figure 5. Maternal administration of βGlcCer had little to no effect on mom and pup ceramides. Tissues were from mothers and offspring in Figure 5, where mothers received daily subcutaneous (s.c.) injections of βGlcCer mix 1 or mix 2. Tissue ceramides from **A)** mom plasma, **B)** pup stomach and **C)** pup lung were analyzed by mass spectrometry. n=8-10 mice per group for a representative experiment of two experiments. *, p<0.05 compared to saline or vehicle group; for significant differences of βGlcCer mix groups versus vehicle groups the crimson red highlighted chain lengths are above the panels.

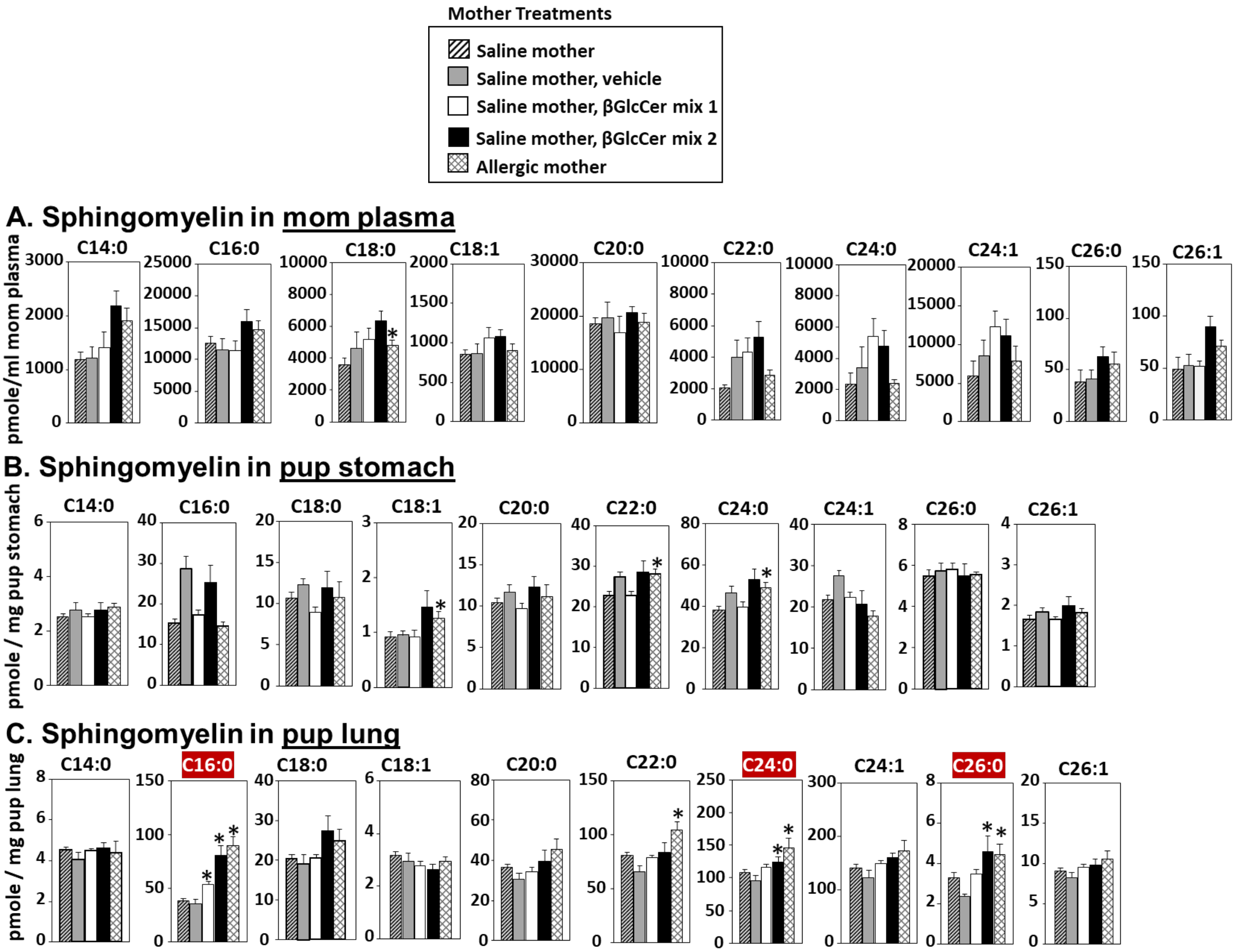

Supplement Figure 6. Maternal administration of βGlcCer had little to no effect on mom and pup sphingomyelins. Tissues were from mothers and offspring in Figure 5, where mothers received daily subcutaneous (s.c.) injections of βGlcCer mix 1 or mix 2. Tissue sphingomyelins from **A)** mom plasma, **B)** pup stomach and **C)** pup lung were analyzed by mass spectrometry. n=8-10 mice per group for a representative experiment of two experiments. *, p<0.05 compared to saline or vehicle group; for significant differences of βGlcCer mix groups versus vehicle groups the crimson red highlighted chain lengths are above the panels.

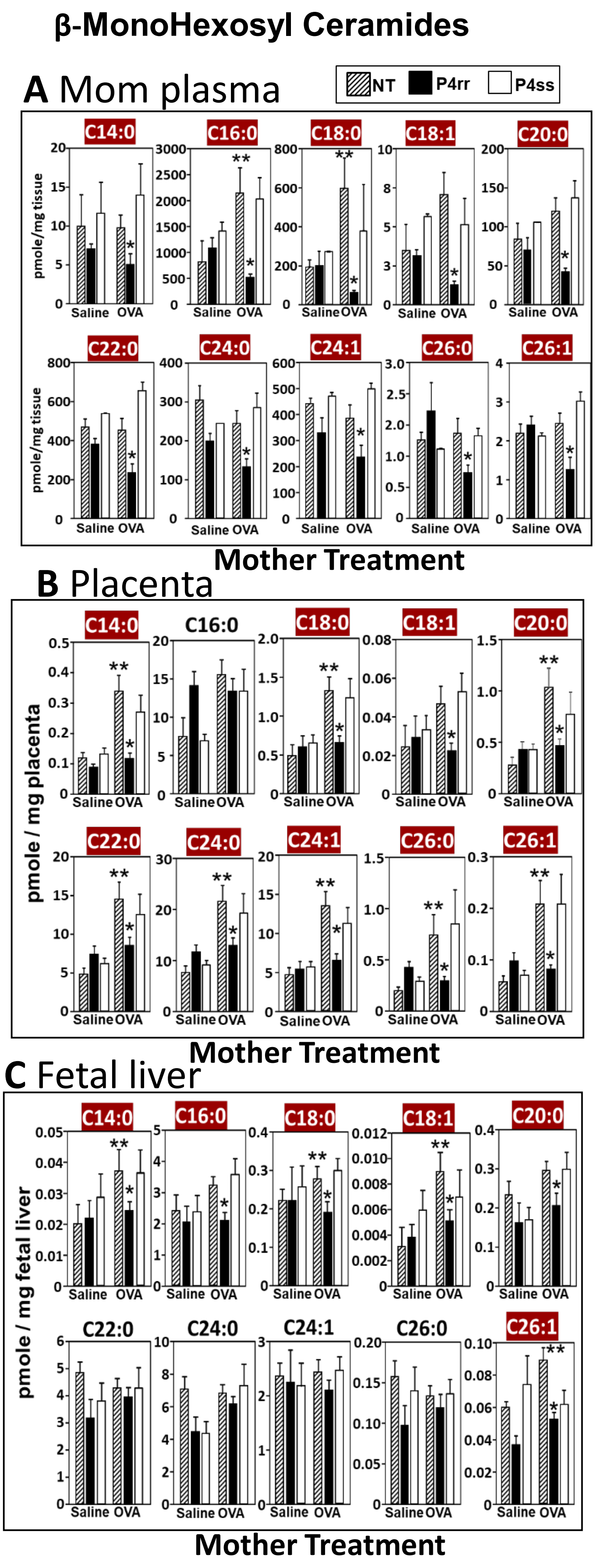

Supplement Figure 7. P4rr decreased β-monohexosyl ceramides in plasma, placenta and fetal liver of allergic mothers. Moms were treated as in Figure 6, maternal plasma, placenta and fetal liver were collected on GD18, and β-monohexosyl ceramides were determined by mass spectrometry. **A)** Mom plasma. **B)** Placenta. **C)** Fetal liver. n=8-10 mice per group for a representative experiment of two experiments. **, p<0.05 compared to pups of NT, saline mother; crimson highlighted chain lengths are above the panels. *, p<0.05 compared to pups of NT, OVA allergic mother; crimson red highlighted chain lengths are above the panels.

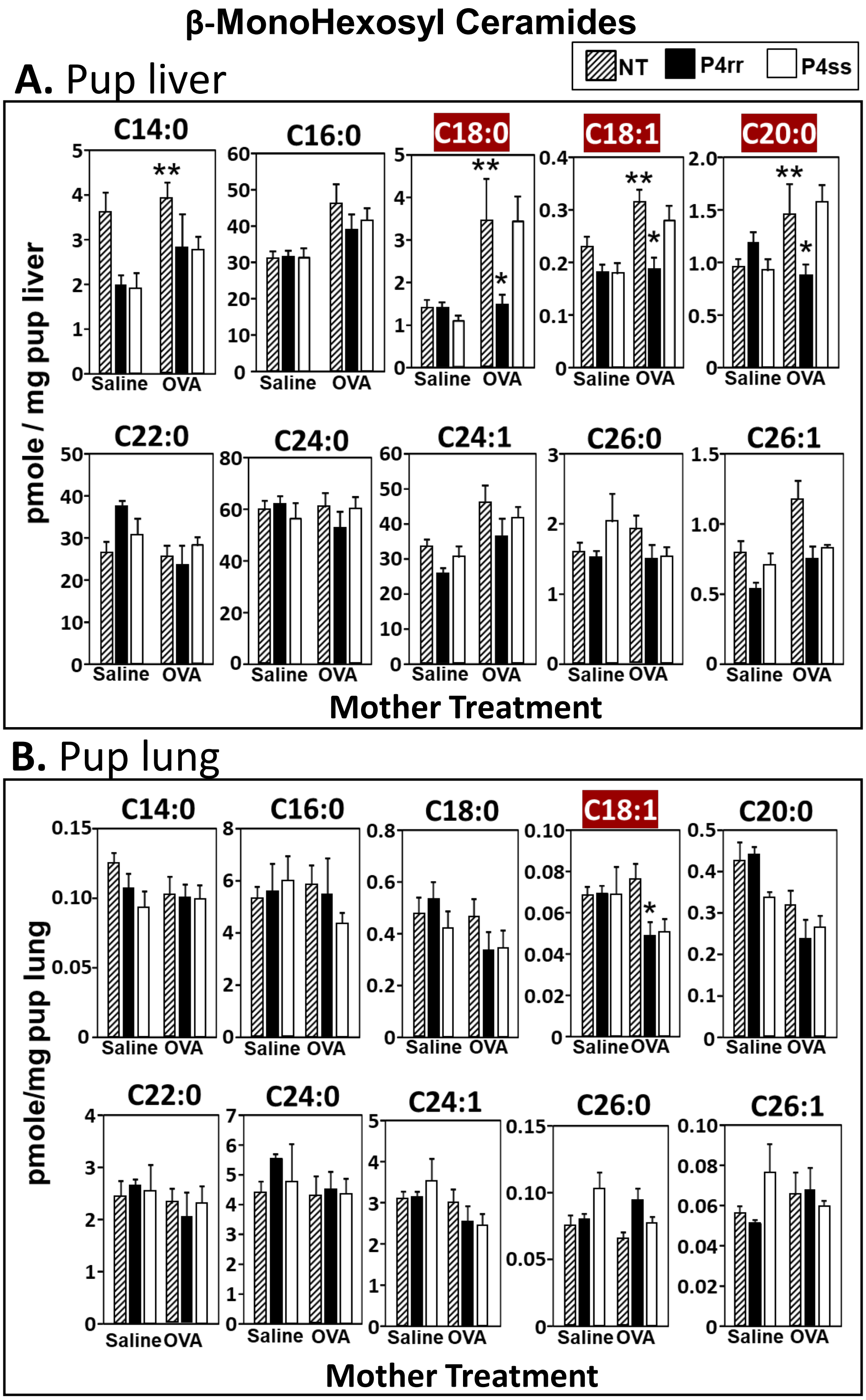

Supplement Figure 8. P4rr decreased β-monohexosyl ceramides in livers and lungs of pups from allergic mothers. Moms were treated as in Figure 6, pup tissues were collected on PND13, and β-monohexosyl ceramides were determined by mass spectrometry**. A)** Pup liver. **B)** Pup lung. n=8-10 mice per group for a representative experiment of two experiments. **, p<0.05 compared to pups of NT, saline mother. *, p<0.05 compared to pups of NT, OVA allergic mother; crimson red highlighted chain lengths are above the panels.

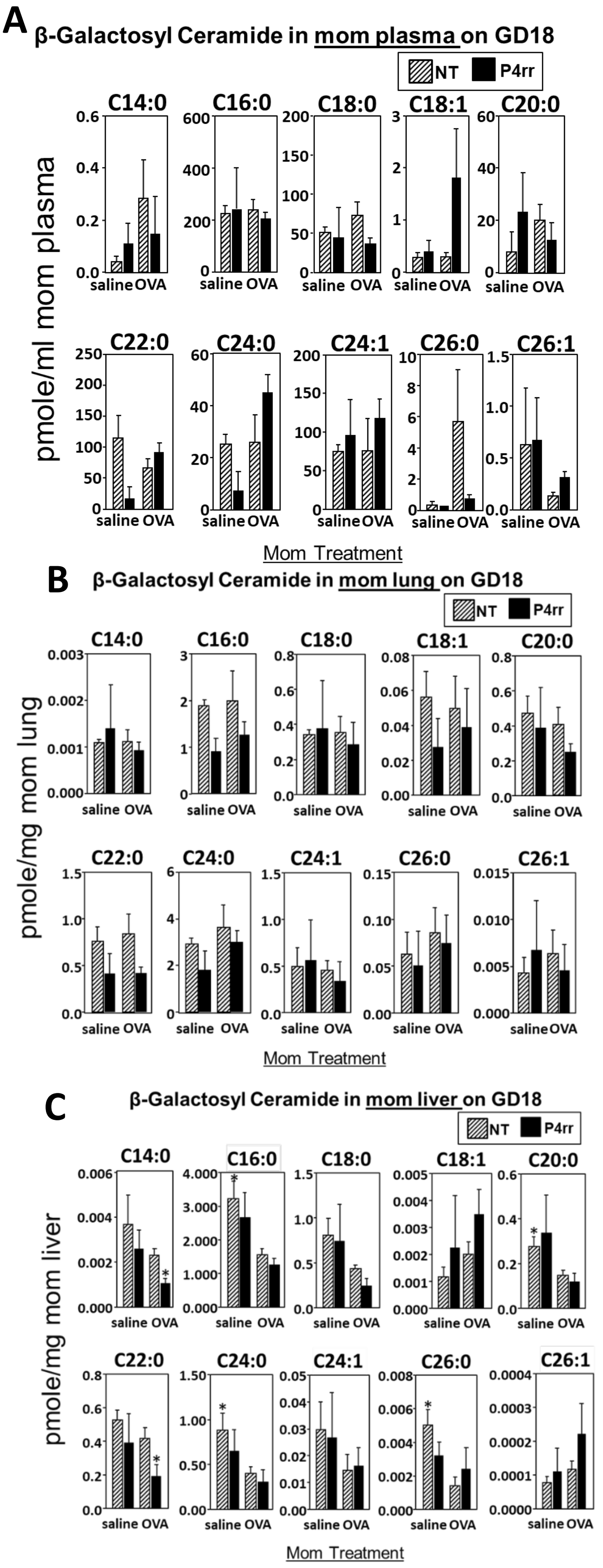

Supplement Figure 9. P4rr did not alter β-galactosyl ceramides in allergic mothers. Moms were treated as in Figure 6, maternal **A)** plasma, **B)** lung and **C)** liver were collected on GD18, and β-galactosyl ceramides were determined by mass spectrometry. n=8-10 mice per group for a representative experiment of two experiments. βGalCer in OVA group was not significantly greater than the saline group. In **C)** *, p<0.05 compared to NT, OVA mother.

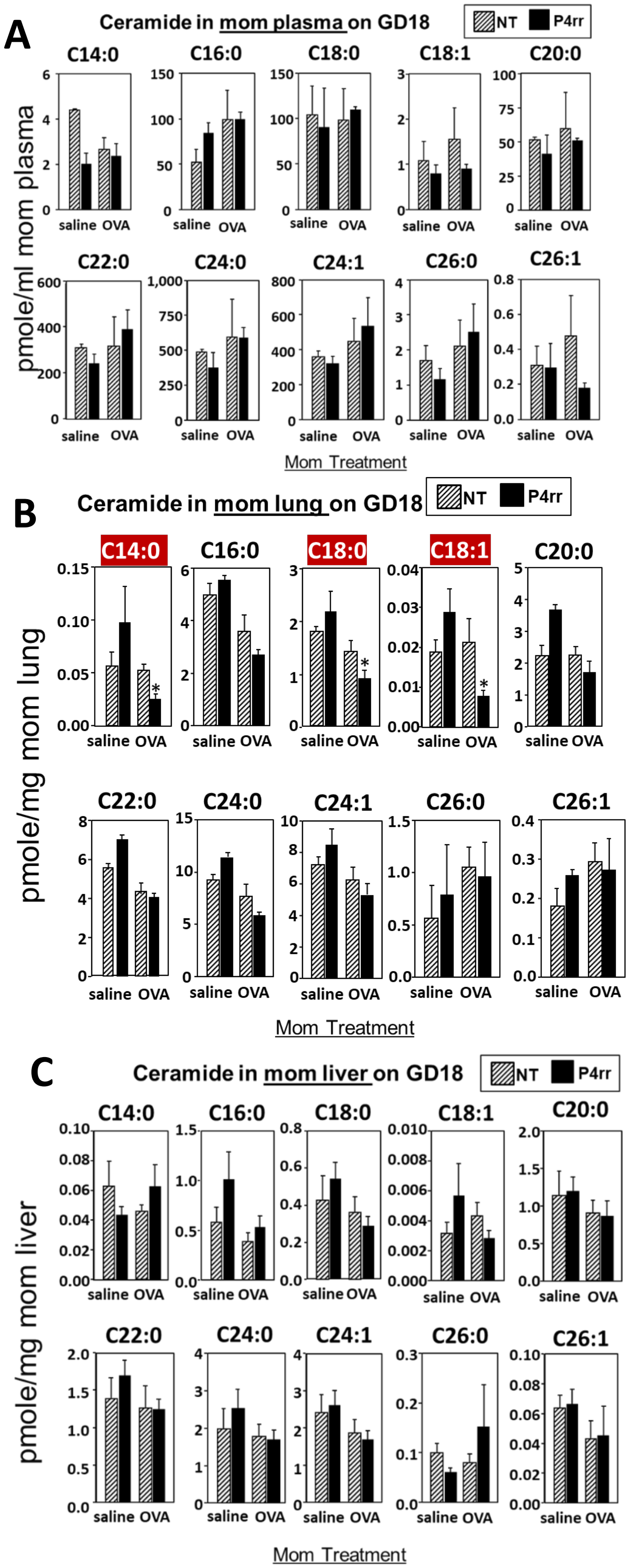

Supplement Figure 10. P4rr had little to no effect on ceramides in allergic mothers. Moms were treated as in Figure 6, maternal **A)** plasma, **B)** lung and **C)** liver were collected on GD18, and ceramides were determined by mass spectrometry. n=8-10 mice per group for a representative experiment of two experiments. *, p<0.05 compared to pups of saline mother; crimson highlighted chain lengths are above the panels.

.

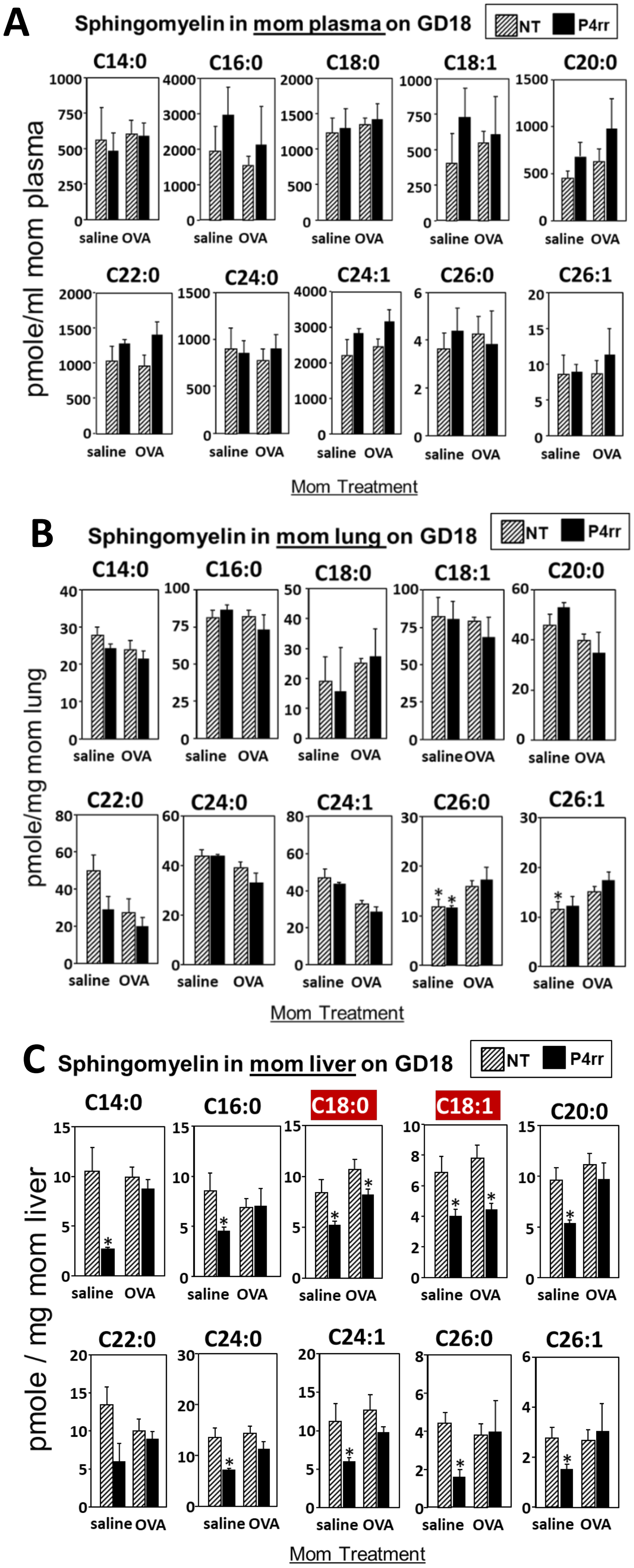

Supplement Figure 11. P4rr had little to no effect on sphingomyelins in allergic mothers. Moms were treated as in Figure 6, maternal **A)** plasma, **B)** lung and **C)** liver were collected on GD18, and sphingomyelins were determined by mass spectrometry. n=8-10 mice per group for a representative experiment of two experiments. **B)** *, p<0.05 compared to NT OVA allergic mother. **C)** *, p<0.05 compared to NT group; highlighted in crimson red are chain lengths for P4rr effect on OVA allergic mothers.

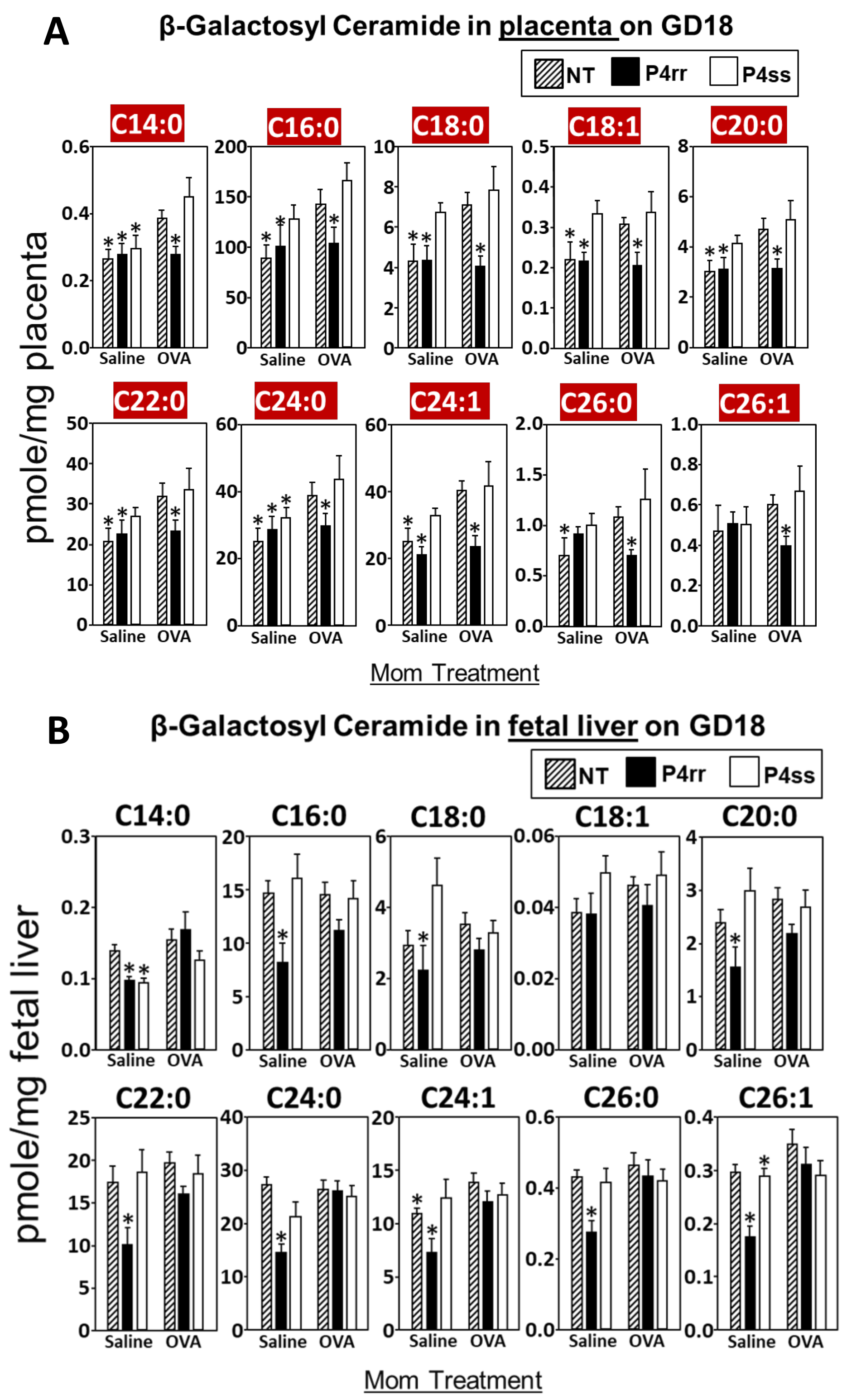

Supplement Figure 12. P4rr reduced β-galactosyl ceramides in placentas and fetal livers of allergic mothers. Moms were treated as in Figure 6, **A)** placentas and **B)** fetal livers were collected on GD18, and β-galactosyl ceramides were determined by mass spectrometry. n=8-10 mice per group for a representative experiment of two experiments. *, p<0.05 compared to NT, OVA allergic mother; for effect of P4rr on OVA group there are crimson red highlighted chain lengths above the panels.

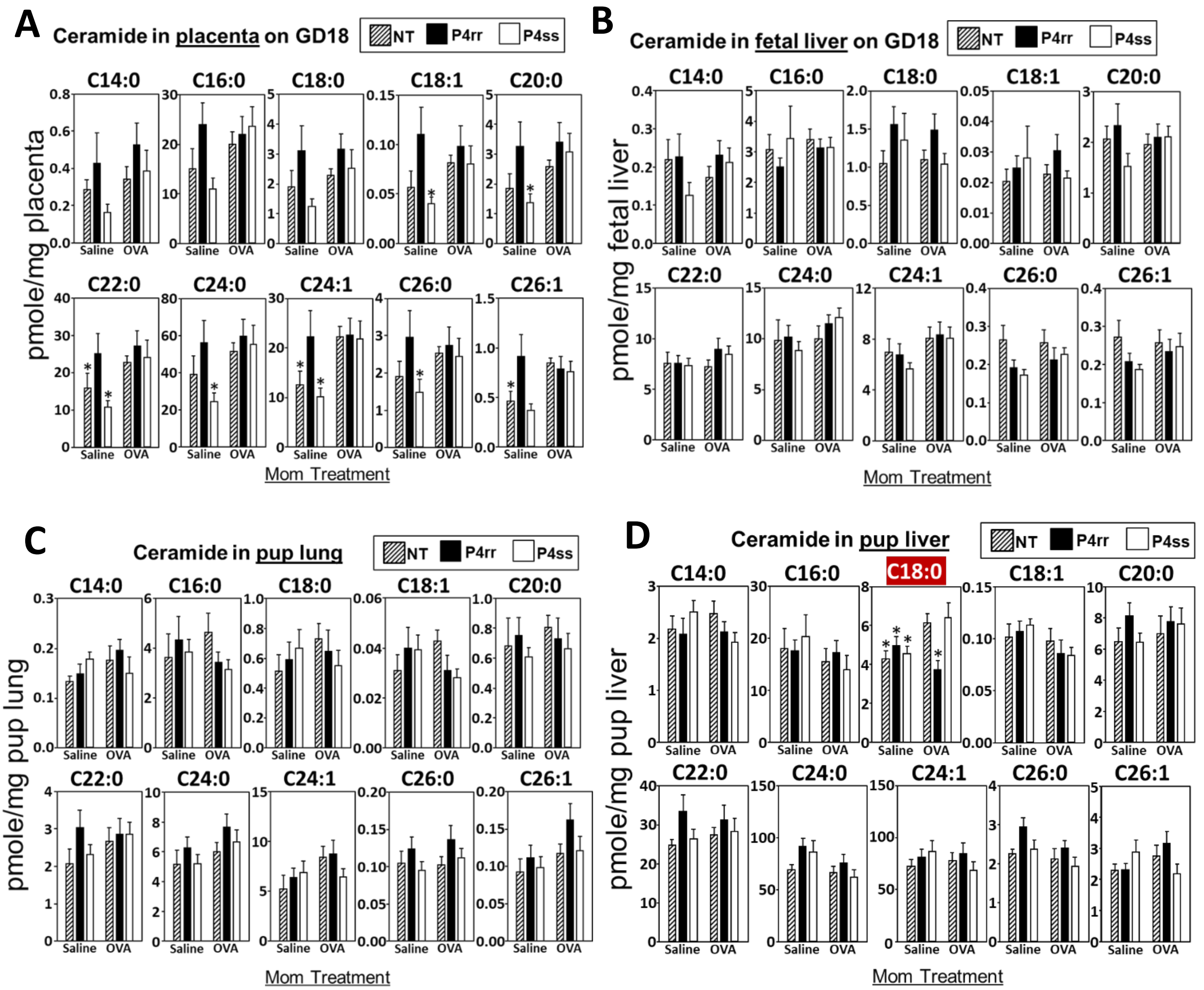

Supplement Figure 13. P4rr had no or little effect on ceramides in offspring of allergic mothers. Moms were treated as in Figure 6, **A)** placentas and **B)** fetal livers were collected on GD18. **C)** pup lungs and **D)** pup livers were collected on PND13. Ceramides were determined by mass spectrometry. n=8-10 mice per group for a representative experiment of two experiments. **A)** *, $p<0.05$ compared to OVA allergic mother, crimson highlighted chain lengths are above the panels. **C)** *, $p<0.05$ compared to NT OVA group; highlighted in crimson red are chain lengths for P4rr effect on OVA allergic mothers..

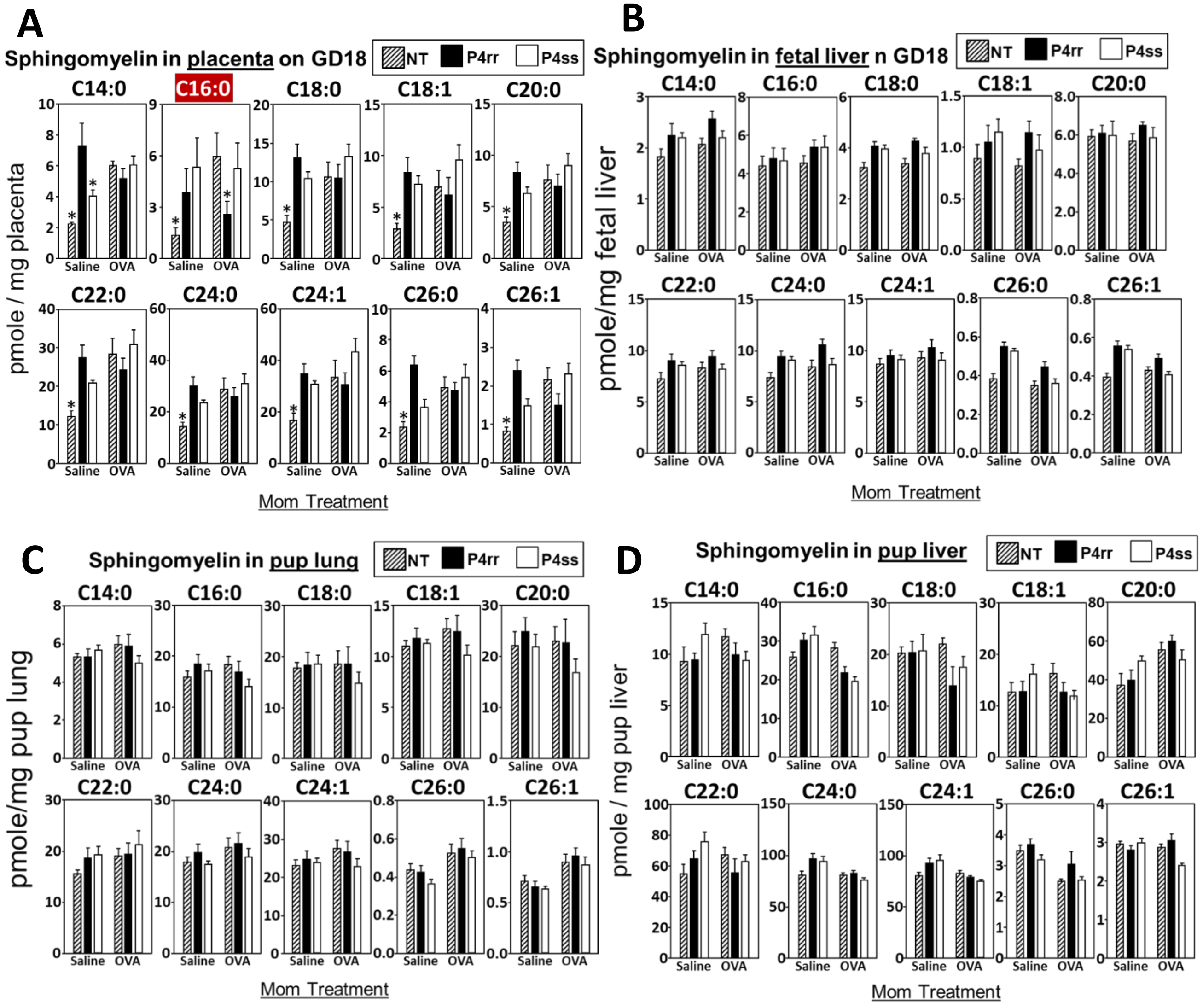

Supplement Figure 14. P4rr did not alter sphingomyelins in offspring of allergic mothers. Moms were treated as in Figure 6, **A)** placentas and **B)** fetal livers were collected on GD18 and **C)** pup lungs and **D)** pup livers were collected on PND13. Sphingomyelins were determined by mass spectrometry. n=8-10 mice per group for a representative experiment of two experiments. **A)** *, p<0.05 compared to NT OVA group; highlighted in crimson red is a chain length for P4rr effect on OVA allergic mothers.

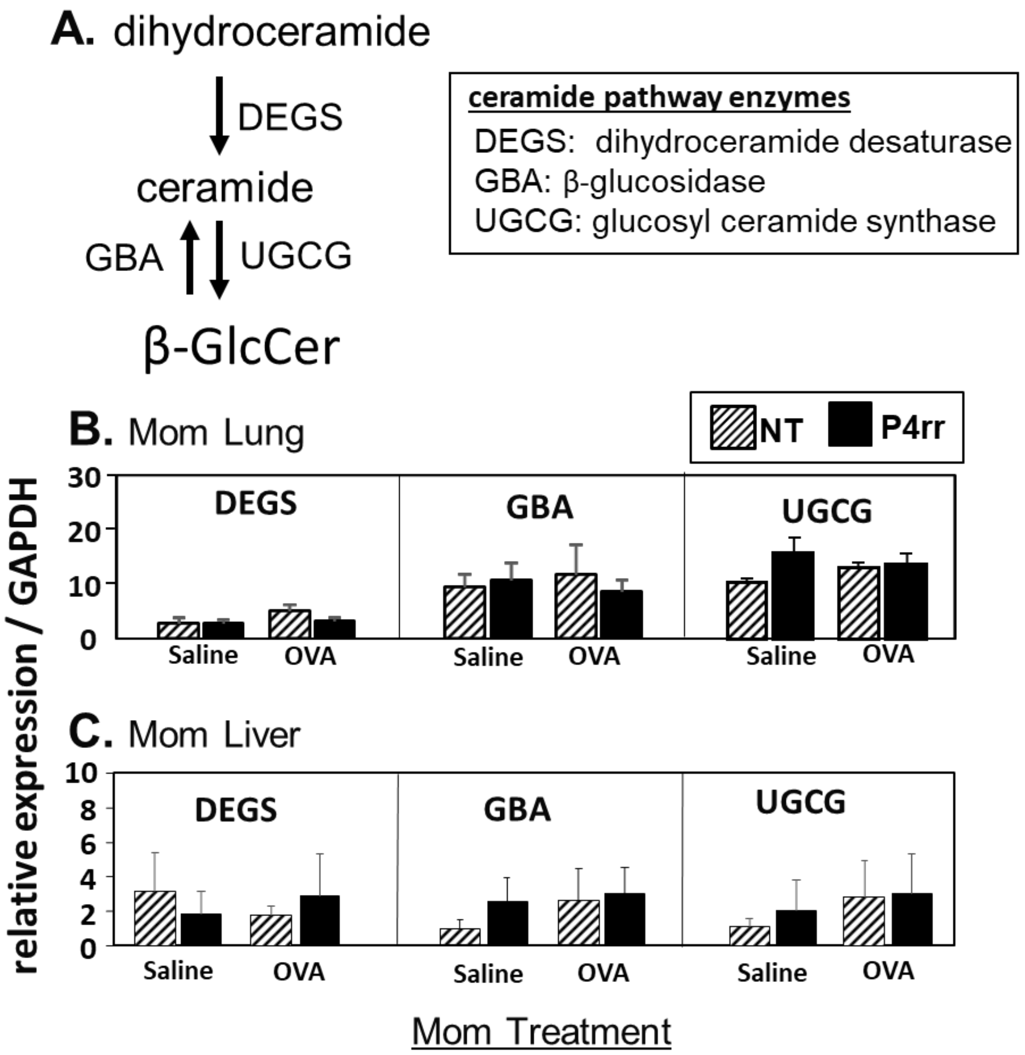

Supplement Figure 15. P4rr did not alter expression of ceramide pathway enzymes in lungs and livers of allergic mothers. A) Ceramide pathway genes. **B,C)** Moms were treated as in Figure 6, lungs and livers were collected on PND13, and enzyme expression was determined by RT-qPCR. n=8-10 mice per group for a representative experiment of two experiments.

.
